# IS*26*-Mediated Transfer of *bla*_NDM–1_ as the Main Route of Resistance Transmission During a Polyclonal, Multispecies Outbreak in a German Hospital

**DOI:** 10.3389/fmicb.2019.02817

**Published:** 2019-12-17

**Authors:** Robert E. Weber, Michael Pietsch, Andre Frühauf, Yvonne Pfeifer, Maria Martin, Dirk Luft, Sören Gatermann, Niels Pfennigwerth, Martin Kaase, Guido Werner, Stephan Fuchs

**Affiliations:** ^1^Department of Infectious Diseases, Robert Koch-Institute, Wernigerode, Germany; ^2^SLK-Kliniken Heilbronn, Institute for Infection Prevention and Clinical Hygiene, Heilbronn, Germany; ^3^German National Reference Centre for Multidrug-Resistant Gram-Negative Bacteria, Department of Medical Microbiology, Ruhr-University Bochum, Bochum, Germany

**Keywords:** antibiotic resistance, carbapenemase, horizontal gene transfer, whole genome sequencing, NDM-1, KPC-2, IS26

## Abstract

One of the most demanding challenges in infection control is the worldwide dissemination of multidrug-resistant (MDR) bacteria in clinical settings. Especially the increasing prevalence of carbapenemase producing Gram-negative pathogens poses an urgent threat to public health, as these enzymes confer resistance to almost all β-lactam antibiotics including carbapenems. In this study, we report a prolonged nosocomial outbreak of various NDM-1-producing *Enterobacterales* species due to clonal spread and cross-species exchange of plasmids and possibly transposons. Between July 2015 and September 2017, a total of 51 carbapenemase-positive isolates were collected from 38 patients and three environmental sources in a single German hospital. Combining molecular typing methods and whole genome sequencing, the metallo-β-lactamase gene *bla*_NDM–1_ was found to be present in 35 isolates of which seven additionally carried the carbapenemase gene *bla*_KPC–2_. Core genome MLST (cgMLST) revealed different clusters of closely related isolates of *Escherichia coli*, *Klebsiella pneumoniae*, *Citrobacter freundii*, *Morganella morganii* or *Enterobacter cloacae* indicating clonal spread. The detailed reconstruction of the plasmid sequences revealed that in all outbreak-associated isolates *bla_NDM–1_* was located on similar composite transposons, which were also very similar to Tn*125* previously described for *Acinetobacter baumannii*. In contrast to Tn*125*, these structures were flanked by IS*26* elements, which could facilitate horizontal gene transfer. Moreover, the identical plasmid was found to be shared by *E. coli* and *M. morganii* isolates. Our results highlight the importance of detailed genome-based analyses for complex nosocomial outbreaks, allowing the identification of causal genetic determinants and providing insights into potential mechanisms involved in the dissemination of antibiotic resistances between different bacterial species.

## Introduction

The rapid spread of antimicrobial resistance (AMR) in nosocomial pathogens poses an urgent threat to patients and public health worldwide. In particular, the increasing incidence of multidrug-resistant (MDR) Gram-negative bacteria that are resistant to fluoroquinolones, third-generation cephalosporins, and carbapenems is of major concern, as these MDR pathogens dramatically limit therapeutic options (Pfeifer et al., 2010; Ruppe et al., 2015; Vasoo et al., 2015). For this reason, bacterial pathogens such as *Acinetobacter baumanii*, *Pseudomonas aeruginosa*, and various *Enterobacterales* species have been identified by the World Health Organization as first priority for research and development of new antibiotics (Tacconelli and Magrini, 2016).

Resistance to third-generation cephalosporins in enterobacterial species is primarily mediated by the acquisition of plasmid-encoded β-lactamases that are characterized by an expansion of the substrate spectrum and are thus referred to as extended-spectrum β-lactamases (ESBL) (Paterson and Bonomo, 2005; Pfeifer et al., 2010). Due to the rapid spread and worldwide dissemination of ESBL-producing *Enterobacterales* and other third-generation cephalosporin-resistant Gram-negative bacteria, carbapenems and other antibiotics of last resort have been increasingly used since the early 1990s (Hawkey and Livermore, 2012). The administration of carbapenems in antimicrobial chemotherapy supported the selection of specific β-lactamases (carbapenemases) that can hydrolyze virtually all β-lactams, including carbapenems, and often resist prominent β-lactam/β-lactamase inhibitor combinations (Queenan and Bush, 2007; Rodriguez-Bano et al., 2018).

In 2009, a new Ambler class B broad-spectrum β-lactamase, the New Delhi metallo-β-lactamase-1 (NDM-1), was described in a *Klebsiella pneumoniae* isolate from a Swedish patient who was previously hospitalized in India (Yong et al., 2009). This enzyme hydrolyzes penicillins, third-generation cephalosporins and carbapenems but not the monobactam aztreonam (Nordmann et al., 2011). Since then, 16 NDM variants have been described in various enterobacterial species from different geographical locations; most of them are encoded by plasmids of distinct Inc types such as IncA/C, IncF, and IncL/M (Khong et al., 2016; Wailan et al., 2016). These plasmids usually co-harbor large collections of genetic AMR determinants, thereby mediating a MDR phenotype (Pfeifer et al., 2010; Nordmann et al., 2011; Bush, 2016). Therapeutics of last resort against MDR and NDM-1-producing Gram-negative bacteria are colistin, tigecycline, and fosfomycin; none of which can be considered ideal in terms of overall efficacy, resistance selection and/or side effects (Nordmann et al., 2011).

Since its first description, NDM-1 has been recorded worldwide in a great diversity of clinical *Enterobacterales* species and has often been associated with a travel history to endemic regions like India, Pakistan, and the Balkan states (Nordmann et al., 2011; Dortet et al., 2014). The potential of *bla*_NDM–1_ to rapidly disseminate between related species of *Enterobacterales* is facilitated by the high genetic mobility of *bla*_NDM–1_ and an association of this resistance determinant with promiscuous plasmids that have a broad host range (Nordmann et al., 2011; Curran and Otter, 2014). As a result of effective horizontal gene transfer (HGT), identical mobile genetic elements (MGEs) occur in different bacteria. However, identical MGEs shared by different bacteria are not necessarily proof of HGT, as they may originate from different sources. The reliable reconstruction of transmission chains is therefore essential to the analysis of multi-species outbreaks, although often difficult and laborious (Pirs et al., 2019). Outbreaks of NDM-1-producing *Enterobacterales* have been reported, as described for the United Kingdom (Fairley et al., 2019), Greece (Politi et al., 2019), Poland (Baraniak et al., 2016), Slovenia (Pirs et al., 2019), Belgium (Heinrichs et al., 2019), Netherlands (Bosch et al., 2017), and most recently Italy (ECDC, 2019). In Europe and more particular in Germany, the prevalence of invasive *Enterobacterales* with resistance to carbapenems is still low, and NDM-1-producing isolates have only been observed occasionally (Becker et al., 2018b). However, data based on the voluntary submission of MDR isolates to the National Reference Centre for Multidrug-resistant Gram-negative Bacteria (NRC) in Bochum, Germany, indicate an increasing number of carbapenemase-producing isolates in Germany over the past years (Robert Koch-Institute, 2018). From 2012 to 2017, the number of NDM-1-producing *Enterobacterales* isolates received from hospitals across Germany rose from 40 to 199. Focusing on all carbapenemase-positive *Enterobacterales*, a total of 1594 isolates were registered in 2017, including 752 Klebsiella pneumoniae, 328 *Escherichia coli* and 199 *Enterobacter cloacae*. The most commonly detected carbapenemase was OXA-48 (*n* = 529), followed by VIM-1 (*n* = 299), NDM-1 (*n* = 199), and KPC-2 (*n* = 148). In the present study, molecular typing methods and whole genome sequencing were combined to trace the prolonged nosocomial outbreak of different NDM-1-producing *Enterobacterales* species back to clonal spread and cross-species exchange of plasmids and possibly transposons.

## Materials and Methods

### Clinical Case Definition

In a German hospital with 18 wards and approx. 1,000 beds, several cases of Gram-negative, carbapenem-resistant bacteria producing NDM-1 and/or KPC-2 (*K. pneumoniae* carbapenemase 2) were detected by PCR in 2015. Two of the first three isolates were obtained from patients with travel history. A clinical case definition based on the successful molecular detection of NDM-1 in *Enterobacterales* was adopted to track a possible spread of these pathogens.

### Bacterial Isolates

As part of the primary diagnostics, species identification and antibiotic susceptibility testing were performed by the hospital’s routine laboratory using the MicroScan WalkAway plus system (Beckman Coulter, CA, United States). Analyses were performed and interpreted according to EUCAST^1^. In case of ambiguous results, carbapenemase detection was conducted using a commercial cassette PCR system considering genes encoding for KPC, NDM, VIM, IMP-1, and OXA-48. Isolates were sent to the NRC to confirm the diagnosis and carbapenemase type. Finally, a total of 51 carbapenemase-positive *Enterobacterales* isolates collected between July 2015 and October 2017 were submitted to the Robert Koch-Institute (RKI) for detailed molecular analyses and strain typing (Supplementary Table 1).

#### Susceptibility Testing

At the RKI, antimicrobial susceptibility testing was repeated by the use of broth microdilution (BMD) according DIN58940 and EUCAST guidelines v9.0 (see text footnote 1). In total, nine antibiotics were tested, including ampicillin, cefotaxime, ceftazidime, cefoxitin, meropenem, gentamicin, amikacin, ciprofloxacin, sulfamethoxazole/trimethoprim, and colistin. Additionally, automated susceptibility testing was performed for piperacillin/tazovactam, aztreonam, cefepime, imipenem, fosfomycin, and tigecycline using the automated test system VITEK^®^ 2 with card AST-N248 (bioMérieux, Nuertingen, Germany).

#### Molecular Typing

##### PCR-based detection of resistance genes

The presence of various β-lactamase genes (*bla*_NDM–like_, *bla*_VIM–like_, *bla*_IMP–like_, *bla*_OXA–48–like_, *bla*_KPC–like_, *bla*_CTX–M–1–2–9group_, *bla*_TEM–like_, *bla*_SHV–like_, *bla*_CMY–like_, *bla*_DHA–like_, *bla*_OXA–1–2–9–10group_, *bla*_PER–like_, *bla*_GES–like_, and *bla*_VEB–like_) was tested by PCR and subsequent Sanger sequencing using previously described primers and protocols (Turton et al., 2006; Grobner et al., 2009; Pfeifer et al., 2011). Additionally, a PCR screening for genes contributing to resistance to fluoroquinolones and aminoglycosides [*aac(6′)Ib-like, qnrA/B/S-like, armA, rmtB*] was performed as described elsewhere (Park et al., 2006; Doi and Arakawa, 2007; Pfeifer et al., 2009). For PCR-based screening of *qnrC* and *qnrD* genes the following primers were used: *qnrC*_fwd 5′-atttccaaggggcaaactg-3′and *qnrC*_rev 5′-aactgctccaaaagctgctc-3′(amplification product 400 bp), *qnrD*_fwd 5′-ttgtgatttttcaggggttg-3′ and *qnrD*_rev 5′- cctgctctccatccaacttc-3′ (amplification product 521 bp).

##### XbaI-macrorestriction and pulsed field gel electrophoresis

Bacterial strain typing was performed by *Xba*I-macrorestriction followed by PFGE. Fragment patterns were interpreted according to the criteria of Tenover et al. (1995).

##### Conjugation experiments

Transfer of β-lactam resistance was tested by broth mating assays with *E. coli* J53 Azi^r^ as the recipient (Clowes and Rowley, 1954). Selection of transconjugants was performed on Mueller–Hinton agar plates that contained sodium azide (200 mg/L) and ampicillin (100 mg/L). Transconjugants were tested for presence of transferred β-lactamase genes and plasmid-mediated quinolone resistance (PMQR) genes by PCR screening. Finally, the plasmid DNA of transconjugants was isolated with the QIAGEN Plasmid Mini Kit according to manufacturer’s instructions (QIAGEN, Hilden, Germany). PCR-based replicon typing (PBRT) was performed using a commercial kit (PBRT KIT, Diatheva, Italy).

##### Hybridization experiments

Plasmid DNA of donor strains and/or transconjugants was isolated using the QIAGEN Plasmid Mini Kit according to manufacturer’s instruction (QIAGEN, Hilden, Germany). Southern hybridization using digoxigenin-dUTP-labeled probes and signal detection using CDP-Star were performed following the manufacturer’s guidelines to localize *bla*_NDM–1_ (Roche Diagnostics Ltd., West Sussex, United Kingdom).

##### S1 nuclease restriction

Plasmid linearization and separation were performed by S1 nuclease restriction of whole genomic DNA (donor strains and transconjugants) combined with PFGE as previously described (Barton et al., 1995).

#### Whole Genome Sequencing and Quality Control

Brain Heart Infusion (BHI) broth was used for the cultivation of bacteria. DNA was extracted from an overnight culture using the DNeasy Blood & Tissue Kit (Qiagen, Hilden, Germany). For quantification of nucleic acids, the Qubit dsDNA HS Assay Kit (Thermo Fisher Scientific, Karlsruhe, Germany) was used in line with the manufacturer’s instructions. Sequencing libraries were generated with the Nextera XT DNA Library Preparation Kit (Illumina, San Diego, CA, United States) and paired-end sequencing was performed using a MiSeq instrument with the 2 × 300 MiSeq v3 reagent kit (Illumina, San Diego, CA, United States). Additionally, different isolates were selected for long-read sequencing via PacBio^®^ Single Molecule Real Time (SMRT) sequencing (isolate 24-16) or Oxford Nanopore Technology (ONT; isolates 20-16, 22-16, 132-16 and 460-16). Isolation of DNA for SMRT and ONT sequencing was performed by using the MagAttract High Molecular Weight DNA Kit (Qiagen, Hilden, Germany) and the Genomic-tip 100/G Kit (Qiagen, Hilden, Germany), respectively, according to the manufacturer’s instructions. ONT sequencing was carried out at the RKI, while SMRT sequencing was conducted by GATC Biotech (Konstanz, Germany). Quality of raw sequence data was checked using FastQC v0.11.5 (Andrews et al., 2012). Additionally, Kraken v0.10.6 was used to verify the taxonomic read classification (Wood and Salzberg, 2014). For reference-based read alignments, raw reads were trimmed using Trimmomatic v0.36 (default parameters) (Bolger et al., 2014).

#### *De novo* Assembly

If available, a *de novo* hybrid assembly was performed based on both long (SMRT/ONT) and short (NGS) reads using Unicycler v0.4.7 with conservative parameters (conservative mode) (Wick et al., 2017). Illumina-only read sets were *de novo* assembled using SPAdes v2.1.3 with default parameters but activated careful mode (mismatch correction) (Bankevich et al., 2012).

#### Phylogenetic Analysis

Multilocus sequence typing (MLST) and core genome MLST (cgMLST) were performed using *de novo* assembled contigs (see section “*De novo* assembly”) and Ridom SeqSphere^+^ v6.0.0 (Ridom; Münster, Germany). The following cgMLST templates were used for *E. coli* and *K. pneumoniae*, respectively: *E. coli* cgMLST^2^ and *K. pneumoniae* sensu lato cgMLST^3^. For *Citrobacter freundii* and *E. cloacae*, an *ad hoc* cgMLST was established according to Ridom SeqSphere^+^ guidelines^4^. For this purpose, *C. freundii* CAV1321 (GenBank: CP011612.1), and *Enterobacter hormaechei* 20710 (GenBank: CP030076.1) were selected as seed genomes. Minimum spanning trees were inferred by ignoring pairwise missing values. Since no stable task templates are available for *Morganella morganii*, phylogenetic relationships were reconstructed on the level of single nucleotide polymorphisms (SNPs). A reference sequence was selected by using *refRank* (Becker et al., 2018a) with a set of 11 complete *M. morganii* genome sequences available at RefSeq (Park et al., 2006). Subsequently, trimmed paired-end reads were aligned to *Morganella morganii* subsp. morganii KT (NC_020418) using our in-house pipeline *batchMap*^5^ as previously described (Halbedel et al., 2018). Alignment of consensus sequences were reduced to variant positions using *SNPFilter* v3.2.3 with default parameters (Becker et al., 2018a) and then used to calculate distance matrices and minimum spanning trees (Geneious v11.1.5).

#### Resistance Gene and Plasmid Replicon Identification

*De novo* assembled contigs were used for both resistance gene and plasmid replicon type identification. For the detection of acquired AMR genes, the ResFinder tool v3.2 was used (Zankari et al., 2012). *In silico* replicon typing was performed with the PlasmidFinder v2.0 (Carattoli et al., 2014). Both tools are available at http://www.genomicepidemiology.org/.

#### Plasmid and Transposon Reconstruction

**FIGURE 1:**
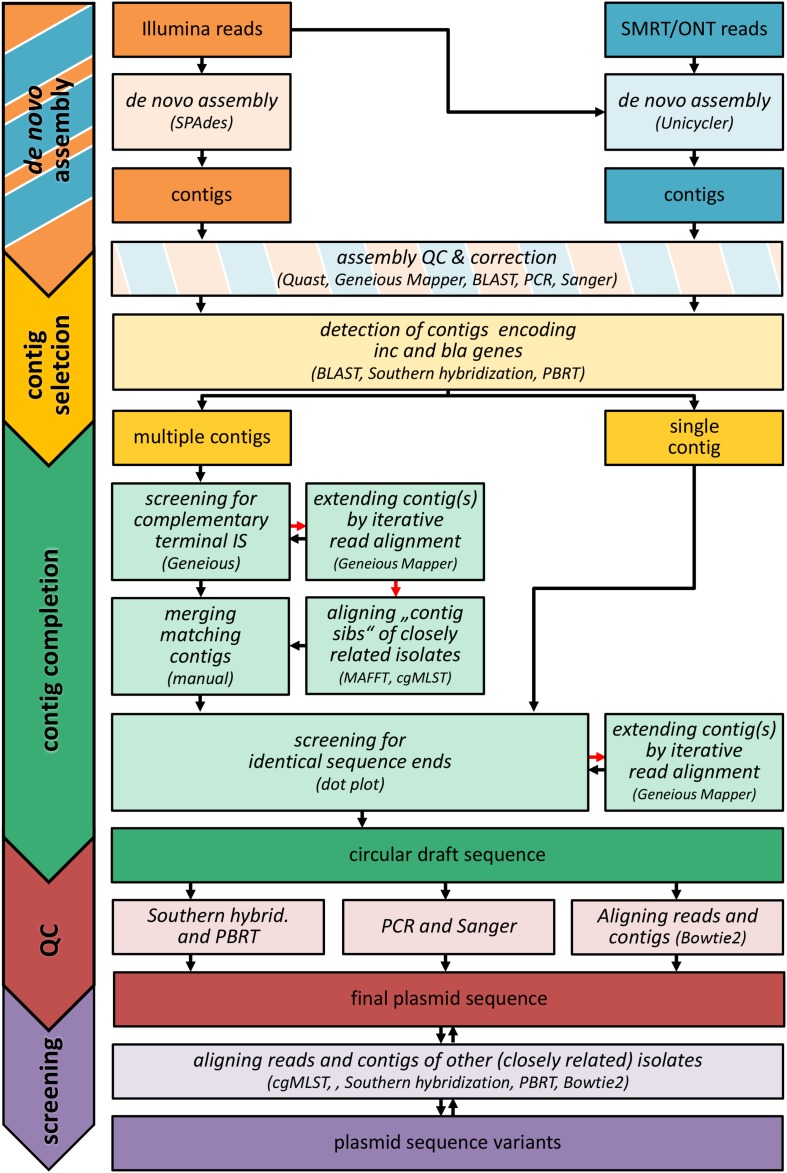
Workflow of plasmid reconstruction. The graphic illustrates the different strategies used for the reconstruction of outbreak-related replicon sequences in this study. The different analyses tasks are shown in italics (bright colored boxes). The respective input and output (reads, contigs, draft and final sequences) is highlighted by boxes with strong colors. If a method could not successfully applied, alternative methods were used (indicated by red arrows). For a detailed description of the reconstruction process, see Supplementary Data Sheet 1. SMRT: PacBio^®^ Single Molecule Real Time (SMRT) sequencing; ONT, Oxford Nanopore Technology; Inc, replication sequences; *bla*, β-lactamase gene; IS, insertion sequence; QC, quality control; PBRT, PCR-based replication typing; contig sib(ling)s, orthologous contigs; cgMLST, core genome MLST.

For the reconstruction of outbreak related MGEs, different strategies have been used (Figure 1). Briefly, *de novo* assembled contigs were screened for *bla_NMD–1_*, *bla_KPC–2_*, and replication sequences using BLAST (Camacho et al., 2009). In order to identify defective assemblies, trimmed Illumina reads were aligned to the selected contigs (Geneious v11.1.5 Mapper with default parameters and fine tuning including up to five iterations) and manually searched for unaligned regions. Unaligned regions were checked using Sanger sequencing (see below) and corrected if necessary (pKP39-T3). In two cases (pEC405-T3, pPS-T1), the detected resistance gene and replication sequences (associated by Southern hybridization and PBRT as described before) were located on separate contigs. These contigs were annotated using a customized database of relevant sequence motifs such as repeats and transposases (Geneious v11.1.5). Corresponding elements (e.g., IS*26* IRR and IRL) located at contig ends were used to merge respective contigs. In several cases (pCF104-T3, pPS-T1, pKP15-T2, pEC744-T5), contigs had to be extended by iterative alignment of trimmed Illumina reads to detect matching elements at their ends (Geneious v11.1.5 Mapper). Merging sites and contig orientation were validated using PCR (see below). To form a circular sequence, (merged) contigs were checked for identical sequence ends. When indicated by complementary motifs (e.g., IS*4321* IRL and IRR), the respective element was added (pKP39-T3, pKP39-T4, pPS-T1, pEC6332-T3). Ring closing sites were validated using Sanger sequencing and/or PCR (see below). To check its integrity, both trimmed Illumina reads and *de novo* assembled (corrected) contigs were aligned to the final sequence (Supplementary Data Sheet 2). Samtools v1.9 (Li et al., 2009) were used to check for proper read pairing (fixmate) and to remove potential PCR duplicates (rmdup). After this, reads were aligned using Bowtie v2.3.5 with stringent parameter settings (–end-to-end –very-sensitive –qc-filter –gbar 30 –no-1 mm-upfront -R 10 –score-min L,0,-0.2) to prevent misleading alignments of very similar but still different reads of repetitive elements from other locations/replicons. Contigs were aligned using Mummer3/Nucmer v3.23 (Kurtz et al., 2004). Sequencing depth was assessed using samtools v1.9 (depth). After the reconstruction, final sequences were used as a reference for isolates that should have the same plasmid based on results from Southern hybridization and PBRT. Respective sets of Illumina reads and *de novo* assembled contigs were aligned using Geneious (Geneious v11.1.5 Mapper with default parameters and fine tuning including up to five iterations). Unaligned regions indicating structural variations (insertions) were corrected based on results from sequence homology searches using BLAST resulting in plasmid variants (pEC405-T3, pEC6332-T7, pCF104-T3). A detailed description of the reconstruction of each plasmid reported in this study can be found in Supplementary Data Sheet 1.

#### Verification of Plasmid Reconstruction

DNA for Sanger sequencing and PCR-based validation (Thermocycler Biometra Tadvanced; Analytic Jena AG, Jena, Germany) of ring closing sites was extracted with the DNeasy Blood & Tissue Kit (Qiagen, Hilden, Germany). Primers were obtained from Integrated DNA Technologies (Leuven, Belgium) (Supplementary Table 2). Sanger sequencing was performed with the BigDye^®^ v3.1 Cycle Sequencing Kit (Thermo Fisher Scientific, Waltham, MA, United States) according to the manufacturer’s instructions. For capillary gel electrophoresis the Applied Biosystems^®^ 3500 series (Thermo Fisher Scientific, Waltham, MA, United States) was used.

## Results

### Epidemiological Setting

**FIGURE 2:**
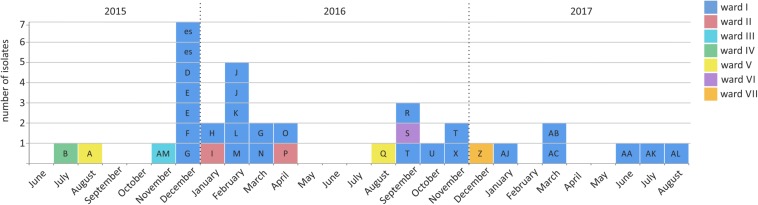
Emergence of NDM-1-producing *Enterobacterales* between July 2015 and October 2017 in a German hospital. In total, 35 NDM-1 producing *Enterobacterales* isolates have been collected from 29 patients (indicated by capital box letters) and two environmental samples (labeled as “es”) across different wards (indicated by box colors).

In July 2015, a carbapenem-resistant *C. freundii* was detected during a routine screening in a German hospital with a capacity of approximately 1,000 beds. The hospital includes 18 hospital wards that cover almost all medical disciplines. As confirmed by the NRC, carbapenem resistance was mediated by the metallo-β-lactamase NDM-1 (gene *bla*_NDM–1_). By an established prospective search including both admission and weekly screenings, 51 carbapenem-resistant *Enterobacterales* producing NDM-1 and/or KPC-2 were detected. These were collected from 38 patients and three environmental sources (rinse water samples) across seven different wards (I-VII) between July 2015 and October 2017. Patients were either colonized (*n* = 31; 36 isolates), infected (*n* = 5; 7 isolates) or both (*n* = 2; 5 isolates). Most patients were primarily hospitalized at the hematologic oncology ward I (Supplementary Table 1). The majority of isolates originated from rectal swabs (*n* = 41). Further isolates were collected from catheter tips (*n* = 2), intraabdominal sources (*n* = 2), urine culture (*n* = 1), wound (*n* = 1), and respiratory tract (*n* = 1) (Supplementary Table 1). Containment and finally cessation of transmission was achieved by extensive staff training and the introduction of substantial organizational, structural and infection control measures. No new cases were observed since the last involved patient (*n* = 45) had been discharged in August 2018 (Figure 2).

### Microbiological Investigation and Molecular Characterization

The *bla*_NDM–1_ gene was detected in 35 isolates from 29 patients and two environmental sources (Figure 2, Supplementary Table 1). NDM-1-positive isolates were identified as *C. freundii* (*n* = 3), *E. cloacae* (*n* = 2), *E. coli* (*n* = 18), *K. pneumoniae* (*n* = 8), *M. morganii* (*n* = 3) and *Providencia stuartii* (*n* = 1). Among these, seven isolates additionally encoded KPC-2. Moreover, we identified 16 *bla*_NDM–1_ negative but *bla*_KPC–2_ positive isolates from 13 patients and one environmental source, that revealed *K. pneumoniae* (*n* = 15) and *E. coli* (*n* = 1) species (Supplementary Table 1). All isolates exhibited resistance to ampicillin, cefoxitin, cefotaxime, ceftazidime, piperacillin/tazobactam, imipenem and meropenem. Resistance to ciprofloxacin and sulfamethoxazole/trimethoprim was detected in 98% of all isolates, and several *K. pneumoniae* were additionally resistant to tigecycline (see Supplementary Table 1 for more details). Resistance to colistin was only measured in intrinsically resistant isolates (*M. morganii* and *P. stuartii*). Besides *bla*_NDM–1_ and *bla*_KPC–2_ additional β-lactamase genes were detected by PCR screening, and these were additionally confirmed by WGS analyses. These included *bla*_TEM–1_ (*n* = 36), *bla*_OXA–1_ (*n* = 32), *bla*_CTX–M–15_ (*n* = 29), *bla*_OXA–9_ (*n* = 15), *bla*_SHV–28_ (*n* = 12), *bla*_CMY–4_ (*n* = 9), *bla*_SHV–1_ (*n* = 7), *bla*_SHV–11_ (*n* = 4), *bla*_CTX–M–1_ (*n* = 2), *bla*_CMY–108_ (*n* = 1), *bla*_OXA–10_ (*n* = 1), *bla*_OXA–2_ (*n* = 1), *bla*_ACC–4_ (*n* = 1), *bla*_TEM–116_ (*n* = 1) and *bla*_ACT–7_ (*n* = 1) (Supplementary Table 1). PFGE analyses revealed different NDM and/or KPC-producing strains of *E. coli* (*n* = 4), *K. pneumoniae* (*n* = 4), *C. freundii* (*n* = 2), *M. morganii* (*n* = 1), *E. cloacae* (*n* = 1), and *P. stuartii* (*n* = 1) (Supplementary Table 1). Conjugation assays combined with S1-nuclease PFGE, PBRT and hybridization experiments showed an association of *bla*_NDM–1_ with plasmids of different sizes and Inc types (Supplementary Table 1).

### WGS-Based Phylogenetic Analyses

**FIGURE 3:**
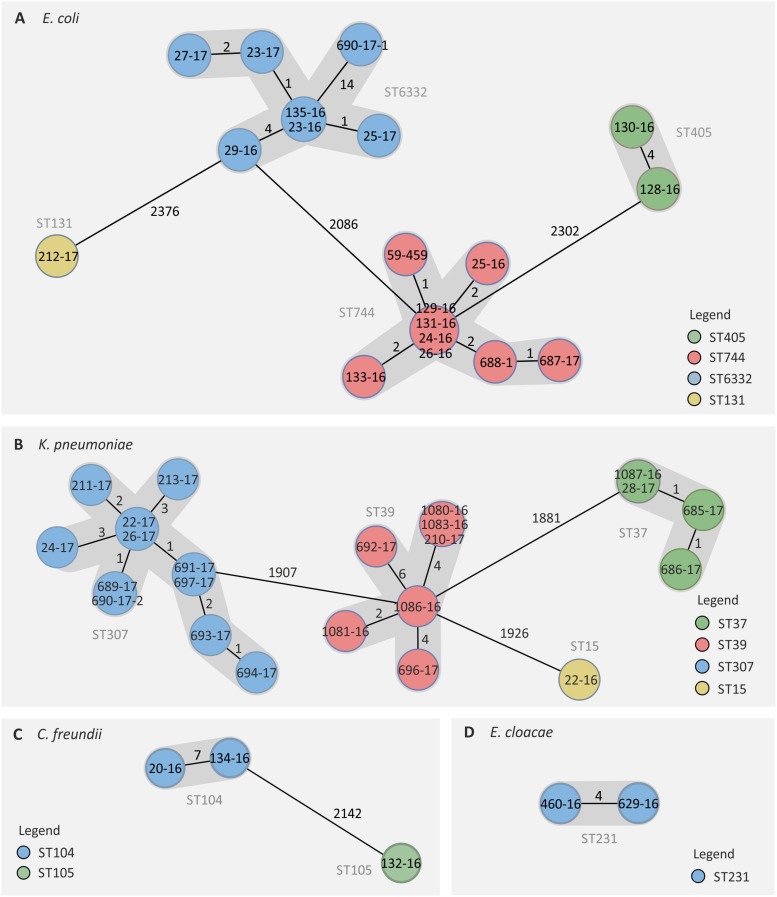
Genetically related clusters of NDM-1-positive *Enterobacterales* based on cgMLST. For *E. coli* **(A)**, *K. pneumoniae* **(B)**, *C. freundii* **(C)**, and *E. cloacae* **(D)** isolates, genetic distances were calculated based on the allelic profiles of 2513, 2159, 4156, and 2385 target genes, respectively (pairwise ignored missing values). The number of different alleles between isolates is shown next to black lines. Please note: the distances displayed are not proportional to the number of differing targets. Isolate names are shown within color-coded circles representing MLST-based sequence types.

cgMLST and SNP-based analyses revealed different clusters of genetically highly related isolates, indicating a clonal spread. The 19 *E. coli* were assigned to three clusters and one singleton that were named according to the respective sequence types (STs) (Figure 3A). The three clusters comprised nine ST744 isolates, seven ST6332 isolates, and two ST405 isolates, respectively, which was consistent with PFGE results (Supplementary Table 1). Within each cluster, isolates did not show more than eight allele differences in the cgMLST (Figure 3A). Three different clusters and one singleton have also been detected for the 22 *K. pneumoniae* isolates hereinafter referred to as ST307 (*n* = 11), ST39 (*n* = 7), ST37 (*n* = 3), and ST15 (*n* = 1) (Figure 3B). Less than eleven different alleles were detected between *K. pneumoniae* isolates of the same cluster (Figure 3B). For three *C. freundii* isolates, the WGS-derived MLST revealed two new STs (ST104 and ST105) of which the two ST104 isolates showed differences in seven alleles only (Figure 3C). Likewise, both ST231 *E. cloacae* isolates were closely related with differences in only four alleles (Figure 3D). Since a cgMLST scheme was not yet available for *M. morganii*, we used a SNP-based analysis to investigate the genetic relationship between those isolates. By this, a maximal difference of six SNPs was found (data not shown).

### Plasmid and Transposon Analyses

**FIGURE 4:**
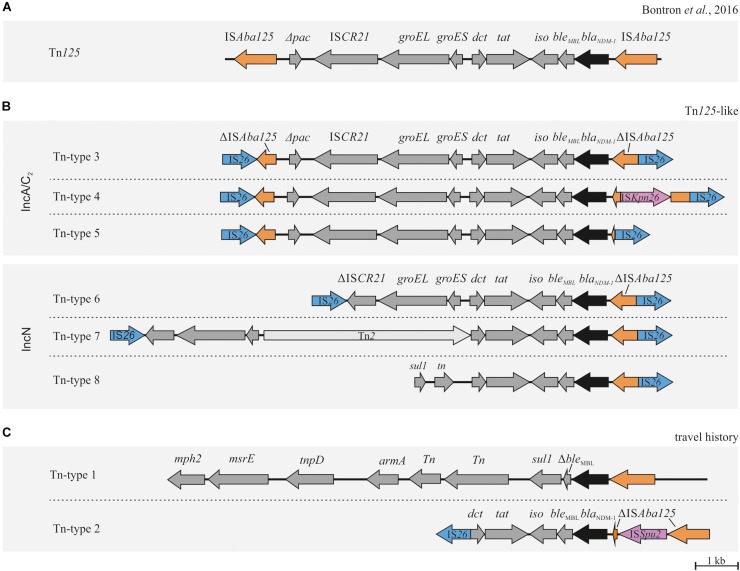
Genetic organizations of *bla*_NDM–1_ associated Tn*125* composite transposons. **(A)** Structure of the genuine Tn*125* composite transposon originally described by Bontron et al. (2016). **(B)** Organization of Tn*125-*like structures in plasmids potentially involved in horizontal transmission. The complete reconstruction of pCF105-T8 failed (Tn-type 8). **(C)** *bla*_NDM–1_ associated structures from isolates of patients with travel history demonstrating a different Tn structure. ORFs and respective orientations are represented by arrows. IS*26* elements are indicated in blue, IS*Aba125* and ΔIS*Aba125* in orange, *bla*_NDM–1_ in black and other ORFs in dark gray.

To assess whether *bla*_NDM–1_ associated MGEs in the different *Enterobacterales* isolates were genetically related or not, WGS data were used to reconstruct the involved genetic elements. For this purpose, data from short- and long-read sequencing were utilized in combination with results of conjugation assays, S1-PFGE-based plasmid size determination and Southern hybridization, thus allowing the complete reconstruction of eleven *bla*_NDM–1_ carrying plasmids present in 34 isolates (Table 1 and Supplementary Image 1). Only for one isolate of this study (132-16 *C. freundii*-ST105), complete reconstruction of the *bla*_NDM–1_ associated replicon (pCF105-T8) failed. A detailed description of the sequence reconstruction processes is given in Supplementary Data Sheet 1. The majority of reconstructed *bla*_NDM–1_ carrying plasmids belonged to incompatibility group IncA/C_2_ (*n* = 8) followed by IncN (*n* = 2) and IncFII/FIB (*n* = 1) (Table 1). Plasmids pCF104-T3 (IncA/C_2_) and pKP15-T2 (IncFII/FIB) originated from isolates of patients with a travel history and showed a unique genetic organization (Figure 4). They were not considered to be involved in local transmission events and therefore not addressed in further details.

**TABLE 1:** Characteristics of NDM-1 encoding plasmids of this study.

**Plasmid**	**Involved species and number of isolates**	**Inc-type (PBRT, WGS)**	**Tn-type**	**Size [kb] (S1 nuclease PFGE)**	**Size [kb] (WGS)**
pPS-T1	*P. stuartii* (1)	IncA/C_2_	1	165	168.7
pEC405-T3	*E. coli* (2)	IncA/C_2_	3	80	88.5
pECl-T3	*E. cloacae* (2)	IncA/C_2_	3	80	94.9
pEC6332-T3	*E. coli* (1)	IncA/C_2_	3	n.d.	115.3
pKP39-T4	*K. pneumoniae* (2)	IncA/C_2_	4	140	156.4
pKP39-T3	*K. pneumoniae* (5)	IncA/C_2_	3	140	155.2
pCF104-T3	*C. freundii* (2)	IncA/C_2_, R	3	170	176.5
pEC744-T5	*E. coli* (9)	IncA/C_2_, R, FIA	5	140	147.5
pKP15-T2	*K. pneumoniae* (1)	IncFII, FIB	2	120	126.5
pEC6332-T6	*E. coli* (2), *M. morganii* (3)	IncN	6	40	>44.5
pEC6332-T7	*E. coli* (4)	IncN	7	45	>49.8
pCF105-T8	*C. freundii* (1)	IncN^1^	8	50	unknown

*^1^confirmed with PCR-based replicon typing (PBRT) only; please note: plasmids pCF104-T3 (IncA/C_2_) and pKP15-T2 (IncFII/FIB) originated from isolates of patients with a travel history and were not included here.*

Interestingly, seven of the eight IncA/C_2_ plasmids (pCF104-T3, pEC744-T5, pEC405-T3, pECl-T3, pEC6332-T3, pKP39-T4, and pKP39-T3) shared a highly conserved backbone structure (Supplementary Image 1). However, they varied in distinct regions containing deletions, additional genes or MGEs and/or multi-replicon/hybrid plasmid elements. These regions were located upstream the antibiotic resistance island ARI-A that mainly affected genes coding for conjugative transfer proteins (Supplementary Image 1). Within ARI-A, we found the *bla*_NDM–1_ gene located on a previously described Tn*125* transposon structure (Bontron et al., 2016). This structure, however, was consistently flanked by two IS*26* elements which were integrated into the adjacent IS*Aba125* elements of Tn*125* (Figure 4). As a consequence, the genuine composite transposon structure of Tn*125* is turned into an IS*26*-dependent composite transposon structure. Owing to this variation, we will refer to it as “Tn*125-*like.” In the seven IncA/C_2_ plasmids three different Tn*125-*like transposon types (Tn-types) had been identified that showed only minor structural variations (Tn-types 3–5) (Table 1 and Figure 4).

In the *E. cloacae* isolate 629-16, we found the IncA/C_2_ plasmid pECl-T3 not only present as plasmid but additionally integrated into the bacterial chromosome (i460-16; potentially flanked by two IS*26* elements). This observation was confirmed by hybridization results (data not shown). The second *E. cloacae* isolate, 460-16, possessed only the chromosomal variant i460-16 flanked by two IS*26* elements as confirmed by ONT.

The *K. pneumoniae* isolates 1080-16, 1081-16, 1083-16, 1086-16, 210-17, 692-17, and 696-17 did not only possess the IncA/C_2_ plasmids pKP39-T4 or pKP39-T3 but also a *bla*_KPC–2_ carrying plasmid designated as pKPC-2. This plasmid (pKPC-2) was found in all NDM-1-positive ST39 *K. pneumoniae* isolates and was also found in other *K. pneumoniae* isolates (ST37 and ST307) (Supplementary Image 2).

In accordance to results from conjugation and hybridization experiments, *bla*_NDM–1_ has also been found to be located on the IncN plasmid pEC6332-T6 in several isolates (Table 1). In pEC6332-T6, the above described IS*26* flanked composite transposon has been integrated upstream of *Hsp20* and a putative transposase (Supplementary Image 1). The involved transposon structure (Tn-type 6) showed only minor deletions compared to the Tn-type 3 found in IncA/C_2_ plasmids (Figure 4). Interestingly, pEC6332-T6 could be detected in both *E. coli* (*n* = 6) and *M. morganii* (*n* = 3) isolates (Table 1) suggesting interspecies transmission. A minor structural variation of pEC6332-T6 (named pEC6332-T7) was present in four *E. coli* isolates (Table 1). This variant is defined by an insertion of *Tn2* in the Tn*125-*like transposon structure of pEC6332-T6, thereby forming a new transposon and plasmid structure (Tn-type 7) (Figure 4).

## Discussion

**FIGURE 5:**
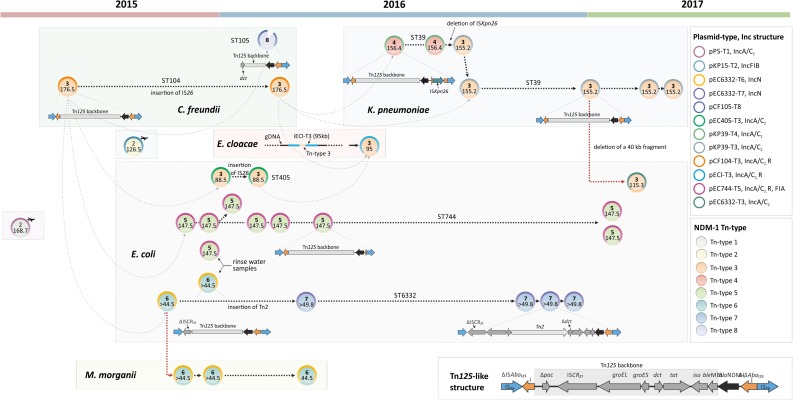
Scheme of NDM-1-encoding plasmids and potential intra-/interspecies transmission pathways between *Enterobacterales* isolates in a German hospital from 2015 to 2017. The illustrated plasmids are horizontally aligned to date of isolation of the respective strain. Plasmid- and Tn-types are represented by color-coded circles and fillings, respectively (see legend on right side). Upper numbers (bold) within plasmid rings highlight Tn-types, while the numbers below show the plasmid size [kb]. The reconstructed transposon structures are schematically illustrated beneath the corresponding plasmids. Directed dotted lines indicate possible vertical (black) and horizontal (red) transmission routes, while gray-shaded lines hypothesize possible transfers of the respective *bla*_NDM–1_ carrying transposon. At the bottom, the Tn*125-*like structure is shown in detail (Tn-type 3). Two *bla*_NDM–1_ carrying plasmids belong to isolates collected from patients with a travel history and, thus not linked to the outbreak (indicated by airplane symbols).

Since carbapenems belong to the most important last resort antibiotics for the treatment of infections caused by MDR Gram-negative bacteria, the emergence of bacteria producing carbapenem hydrolysing enzymes is of particular interest. KPC-2 producing strains, especially *K. pneumoniae*, have been frequently reported in Europe; whereas the number of reports on NDM-1 producers is much smaller (ECDC, 2019; Fairley et al., 2019; Hernandez-Garcia et al., 2019). Hospital-associated infections caused by NDM-1-producing *Enterobacterales* have recently been described in Spain, England, and Italy (ECDC, 2019; Fairley et al., 2019; Hernandez-Garcia et al., 2019). In Germany, screening for “carbapenem-resistant *Enterobacterales* and *A. baumanii* including NDM-1-producing bacteria” is currently only recommended for patients who were previously admitted to hospitals in “high-risk countries” such as India, Pakistan and the Balkan states (KRINKO, 2012). Colonization and infections with carbapenem-resistant *Enterobacterales* and *A. baumanii* have been notifiable since 2016 in Germany. For about half of the notified cases accompanying microbiological data are available (2017: *n* = 1488/2633; 2018: 2061/3794). NDM-1 accounted for 13% (*n* = 56) in 2017 and 4% (*n* = 23) in 2018 of all *Klebsiella* spp. isolates. Moreover, 9% (*n* = 17) of *E. coli* isolates in 2017 and 5% (*n* = 15) in 2018 were positively tested for *bla*_NDM–1_. Considering a substantial underreporting of the number of NDM-1 cases, we could still assume and extrapolate that during the time of the present study 2015–2017, *bla*_NDM–1_ was a rare carbapenemase type in Germany. Therefore, the spatial and temporal clustering of NDM-1-positive isolates in a single hospital – as described in this study – strongly pointed to possible transmissions across different bacterial species. To verify this, we complemented and combined results from traditional typing methods such as PFGE and MLST with WGS data from NGS and ultra-long sequencing technologies. This combination made it possible to track and resolve different (potential) transmission routes (Figure 5).

Both PFGE and cgMLST analyses identified consistent clusters of genetically highly related isolates (e.g., *E. coli* ST744, ST6332 or *K. pneumoniae* ST307, Figure 3) suggesting a clonal spread of these strains (most likely by person-to-person transmission) and, thus, the vertical transmission of NDM-1. Isolates belonging to the largest clonal clusters formed by *E. coli* ST744 (*n* = 9) and ST6332 (*n* = 7) were detected almost within the entire screening period. Clonal spread of NDM-1-positive species have been described in various parts of the world mainly caused by *K. pneumoniae* and *E. cloacae* (Jin et al., 2015; Mahida et al., 2017; Bocanegra-Ibarias et al., 2019; Fairley et al., 2019; Monteiro et al., 2019). Nosocomial outbreaks caused by *K. pneumoniae* ST39, ST37, and ST307 have also been reported worldwide (Belbel et al., 2014; Zhu et al., 2016; Bocanegra-Ibarias et al., 2019). Moreover, the here detected *K. pneumoniae* ST37 and ST307 in combination with KPC-2 production were classified as “high-risk clones” (Yan et al., 2015; Villa et al., 2017).

Isolates of different NDM-1-producing species (*E. coli* and *M. morganii*) collected from different patients shared the identical plasmid (pEC6332-T6) suggesting horizontal gene spread as the most probable route of transmission. Several studies already reported a horizontal transfer of *bla_NDM–1_* and/or other plasmid encoded resistance determinants between different enterobacterial species linked to nosocomial outbreaks (Borgia et al., 2012; Bosch et al., 2017; Schweizer et al., 2019). However, using traditional molecular typing methods we were able to locate *bla*_NDM–1_ on plasmids of various sizes and Inc types (Supplementary Table 1 and Figure 5). The limited resolution of techniques used beforehand hardly allowed a reliable reconstruction of possible transmission events via smaller MGEs such as transposons. It makes whole genome sequencing including long read sequencing technologies indispensable to resolve such scenarios adequately (Salipante et al., 2015). In the present study, however, molecular typing information proved to be extremely helpful, if not essential, for the reconstruction of replicon sequences (see Supplementary Data Sheet 1).

In this study, *bla*_NDM–1_ was mainly associated with IncA/C_2_ plasmids. These broad host range plasmids have been associated with transcontinental spread of *bla*_NDM–1_ in Asia, North America, Africa and Europe and have frequently been described to carry various resistance determinants including β-lactamase genes like *bla*_NDM–1_ and *bla*_CMY–2_ (Fernandez-Alarcon et al., 2011; Harmer and Hall, 2015; Pietsch et al., 2018; Rozwandowicz et al., 2018).

Interestingly, in 2017 an outbreak of NDM-1-producing *K. pneumoniae* was described in a Dutch hospital with an inter-species transfer of a *bla*_NDM–1_ carrying IncA/C_2_ plasmid (Bosch et al., 2017). The sequence of this plasmid shared significant sequence similarities with the plasmids pKP39-T3 and pKP39-T4 described in the present study (data not shown). This similarity may be attributed to the fact that IncA/C_2_ plasmids usually possess a conserved backbone structure including characteristic core regions such as the conjugative transfer region (*tra*) and genes for plasmid maintenance (Harmer and Hall, 2015). In line with these findings, we found a conserved backbone structure for all IncA/C_2_ plasmids analyzed in this study (Supplementary Image 1). However, several structural variations were found in close proximity to the antibiotic resistance island ARI-A such as the acquisition of additional genetic elements and/or the loss of *tra* genes in pCF104-T3, pEC744-T5, pEC405-T3, pECl-T3, and pEC6332-T3 (Supplementary Image 1). Broth mate conjugation assays were not successful for the most IncA/C_2_ plasmids of this study (see Supplementary Table 1). Fernandez-Alarcon et al. (2011) previously described rearrangements in *tra* regions of several plasmids carrying IS*Ecp1*-*bla*_CMY–2_ which might correlate with transmission deficiency. Due to their high sequence variability it is unlikely that all IncA/C_2_ plasmids originated from a common source.

A more detailed analysis of the plasmids of the present study showed that *bla*_NDM–1_ is located in a conserved sequence within the ARI-A of IncA/C2 and various sites of IncN plasmids, respectively (Figure 4 and Supplementary Image 1). This structure has been previously described as Tn*125* (Bontron et al., 2016). In 2016, Bontron et al. (2016) demonstrated that the Tn*125* composite transposon can efficiently transpose within *A. baumanii* which most likely serves as an intermediate reservoir for the *bla*_NDM–1_ gene. The genetic organization of Tn*125* is defined by terminal IS*Aba125* elements that have been shown to be functional for transposition (Bontron et al., 2016). It is assumed that the high prevalence of IS*Aba125* in *Acinetobacter* spp. genomes indicates frequent transposition events, the presence of HGT, a replicative transposition mechanism, and the ability of IS*Aba125* to expand within genomes (Bontron et al., 2016). In the present study we found this genuine Tn*125* structure slightly changed by the integration of IS*26* into each IS*Aba125* element (Figure 4). The *bla*_NDM–1_ gene described in almost all isolates in our study was found to be located within Tn*125*-like structures supporting the theory of a common origin of *bla*_NDM–1_ (Figure 4). In 2015, a very similar transposon associated with *bla*_NDM–4_ was identified in *E. coli* isolates from a transmission event in a Chinese hospital (Zhang et al., 2018). Similar to our findings IS*26* inserted into IS*Aba125* upstream of *bla*_NDM–4_, however, its orientation was not reverse to *bla*_NDM–4_ and a duplication of IS*26* was present in IS*Aba125*. Moreover, an IS*26* element (in reverse orientation to *bla*_NDM–4_) was found instead of a downstream IS*Aba125*. The authors hypothesized that the two flanking IS*26* have the potential to form a mobile composite transposon. Supporting this, IS*26* elements are known to be crucial for the acquisition and dissemination of resistance genes and have also been reported to be involved in transposon building (Argudin et al., 2017; Montana et al., 2017). Thus, IS*26* might be responsible for the transposition of composite transposons between different plasmids. Likewise we hypothesize that IS*26* plays a key role in the mobilization of the identified *bla*_NDM–1_-IS*26* composite transposon structures. We found only minor structural variations of the IS*26*-flanked Tn*125* composite transposon among our *bla*_NDM–1_ carrying IncA/C and IncN plasmids (IncA/C_2_ and IncN) that might indicate that these transposons were exchanged across different plasmids. Based on the detected variations mainly occurred in regions of IS*26* in IS*Aba125* we defined six different Tn-types (Figure 4 and Supplementary Image 1). Based on these variations we hypothesized a certain transposon flux with Tn-type 3 as a driving force.

## Conclusion

In conclusion, we depicted different transmissions of NDM-1-encoding MGEs within isolates of various bacterial species present at the same time in a single hospital. A clonal spread of different NDM-1-producing *Enterobacterales* strains was clearly supported by cgMLST analyses for *E. coli, K. pneumoniae, C. freundii*, and *E. cloacae*. Furthermore, there are clear indications of inter-species transmission of IncN plasmids carrying *bla*_NDM–1_, and potential transmission of mobilized Tn*125*-like transposons with *bla*_NDM–1_ into different plasmids or into the chromosome. Reliable reconstruction of these complex transmission routes was only possible by a combination of traditional plasmid typing methods and hierarchical bioinformatics analyses using NGS and SMRT/ONT data. An assumption or molecular evidence of a clonal spread of resistant strains is essential to initiate infection control and prevention measures. On the other hand, multispecies outbreaks often go undetected. The situation described in this study only became clear through the comparatively high prevalence of a rare carbapenemase gene. We therefore assume a certain underestimation of “plasmid- and/or transposon-driven outbreaks” in general.

## Data Availability Statement

Raw reads datasets generated for this study can be found in the European Nucleotide Archive (https://www.ebi.ac.uk/ena; Study accession number: PRJEB34353). Plasmid assemblies can be found in GenBank under the following accessions: MN657241 (pCF104a-T3), MN657242 (pEC405a-T3), MN657243 (pEC744-T5), MN657244 (pEC6332-T3), MN657245 (pEC6332-T6), MN657246 (pEC6332-T7), MN657247 (pECl-T3), MN657248 (pKP15-T2), MN657249 (pKP39-T3), MN657250 (pKP39-T4), MN657251 (pKPC-2), and MN657252 (pPS-T1).

## Author Contributions

GW, SG, YP, and SF conceptualized and designed the study. MM and DL collected the isolates and metadata, and primary diagnostics and strain characterization. MK, NP, YP, and SG were involved in confirmatory diagnostics and strain characterization. AF was involved in WGS sequencing. RW, MP, AF, SF contributed to the data curation and analyses. RW, MP, SF, YP, and GW wrote the manuscript. All authors edited and reviewed the manuscript.

## Conflict of Interest

The authors declare that the research was conducted in the absence of any commercial or financial relationships that could be construed as a potential conflict of interest.

## Footnotes

1 www.eucast.org

2 https://enterobase.warwick.ac.uk/species/index/ecoli

3 www.ridom.de/seqsphere/u/Task_Template_Sphere.html

4 www.ridom.de/seqsphere/u/Tutorial_for_Ad_hoc_cgMLST_Scheme.html

5 https://gitlab.com/s.fuchs/batchMap
