# Supplementary figures and images for: IS26-Mediated Transfer of blaNDM–1 as the Main Route of Resistance Transmission During a Polyclonal, Multispecies Outbreak in a German Hospital
Source: Front Microbiol. 2019 Dec 17;10:2817. doi: 10.3389/fmicb.2019.02817 (PMC6929489; doi:10.3389/fmicb.2019.02817)

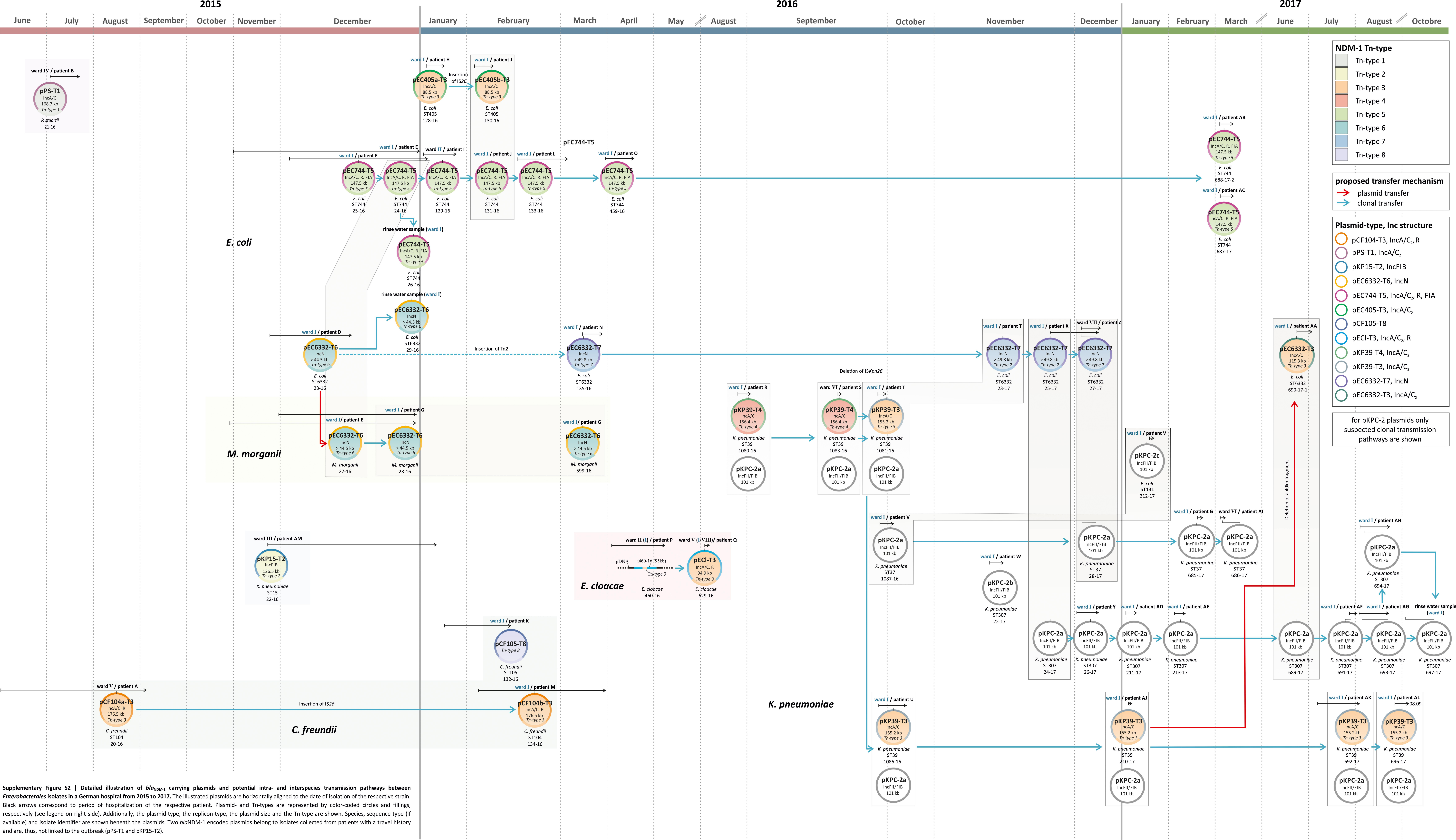

Supplement: Supplementary file 2 [file Image_2.pdf]
