# Supplementary material for: IS26-Mediated Transfer of blaNDM–1 as the Main Route of Resistance Transmission During a Polyclonal, Multispecies Outbreak in a German Hospital
Source: Front Microbiol. 2019 Dec 17;10:2817. doi: 10.3389/fmicb.2019.02817 (PMC6929489; doi:10.3389/fmicb.2019.02817)
Supplement: Supplementary file 3 [file Data_Sheet_1.pdf]

Supplementary Data Sheet 1

1. WGS-based reconstruction of MGEs ... 1
1.1 Illumina-only reconstructions ... 1
1.1.1 Plasmids pKP39-T3 and pKP39-T4 ... 1
1.1.2 Plasmids pEC6332-T6 and pEC6332-T7 ... 2
1.1.3 Plasmids pEC405a-T3 and pEC405b-T3 ... 3
1.1.4 Plasmid pPS-T1 ... 4
1.1.5 Plasmids pKPC-2a, pKPC-2b, and pKPC-2c ... 4
1.1.6 Plasmid pEC6332-T3 ... 5
1.2 NGS- and SMRT/ONT-based reconstructions ... 5
1.2.1 Plasmid pEC744-T5 ... 5
1.2.2 iECl-T3 and Plasmid pECl-T3 ... 6
1.2.3 Plasmid pCF104a-T3 ... 6
1.2.4 Plasmid pKP15-T2 ... 7

1. WGS-based reconstruction of MGEs

The complexity of analysed sequences plays a crucial role in their reference-free (*de novo*) reconstruction based on short read data. DNA and especially plasmid DNA contains repetitive elements that occur multiple times at different locations of the same or coexisting replicons. If these elements are too long to be spanned by a single read or read pair, the resulting assembly path will consist of contigs with multiple in- and outbound connections. Since this cannot be solved any further, the assembly will remain incoherent comprising individual contigs with unknown order and orientation to each other. To create a draft and final sequences we thus used different strategies (Figure 1).

1.1 Illumina-only reconstructions

1.1.1 PlasmidspKP39-T3 and pKP39-T4

Starting with isolate **1086-16** (*K. pneumoniae* ST39), *bla*$_{NDM-1}$ and IncA/C_2 replication sequences were identified on the same contig obtained from *de novo* assembly. Both the contig length (153kb) and Inc type correspond to results from hybridization and PBRT experiments. Manual inspection of reads aligned to the contig sequence, revealed a misassembled region flanked by IS*26* elements upstream of ΔIS*Aba125* which has been corrected using Sanger sequencing. Additionally, IS*4321* IRL and IS*4321* IRR were identified at the contig ends, respectively, indicating the presence of

IS*4321*. The existence of IS*4321* has been validated using PCR resulting in a circular sequence of 155,176bp. Ring closing was validated using PCR. The final sequence, named **pKP39-T3**, was additionally validated by aligning NGS reads and assembled contig (Figures S3 and S4[1]).

Based on cgMLST considering 2159 core genes, the *K. pneumoniae* ST39 isolates **1081-16**, **1083-16**, **1086-16**, **210-17**, **692-17**, and **696-17** are very closely related to **1086-16** (different in less than six alleles) (Figure 2). Additionally, these isolates share a similar β-lactamase gene pattern indicating a common set of accessory genes (Table S1). Based on results from Southern hybridization and PBRT experiments we suggested **pKP39-T3** to be shared by those isolates. To test this hypothesis, NGS reads and assembled contigs of the respective isolates were aligned to the sequence of **pKP39-T3** (Figures S3 and S4[1]). By this the full plasmid was recovered for isolates **210-17**, **692-17**, and **696-17** with less than 2 SNPs. In contrast aligning NGS reads and assembled contigs of strain **1081-16** to **pKP39-T3** resulted in an unmatched region within *InsE* (n=1) that could not be resolved using Sanger sequencing yet. After aligning assembled contigs of strain **1083-16** to **pKP39-T3**, several mismatches were detected in the transition region of two contigs within ΔIS*Aba125* (data not shown). The respective contig ends encoded for IS*Kpn26* IRR and IS*Kpn26* IRL, respectively, indicating the presence of IS*Kpn26*. After validation using Sanger sequencing both contigs were merged considering IS*Kpn26*. NGS reads seamlessly aligned to the corrected sequence supporting its genetic organization (Figure S5[1]). This sequence variant of pKP39-T3 has been referred to as **pKP39-T4** (156,376bp). By aligning respective NGS data, presence of **pKP39-T4** has been confirmed in strain **1080-16**, too (Figures S5 and S6[1]).

1.1.2 Plasmids pEC6332-T6 and pEC6332-T7

Based on NGS data of strain **23-16** (*E. coli* ST6332), we identified a single contig (44kb) encoding NDM-1 and IncN which is in accordance with results from hybridization and PBRT experiments. Identical regions at both contig ends indicated a ring-closed structure which was confirmed by PCR. Additionally, an N-rich repeat region was detected in close proximity to the IncN replicon sequence. Based on PCR this region consists of approximately 1,400bp but could not be fully recovered using Sanger sequencing. We therefore consider the size of **pEC6332-T6** to be at least 44.5kb (Figure S1[1]).

Based on cgMLST analyses considering 2513 core genes, strains **28-16**, **29-16, 135-16**, **23-17**, **25-17**, and **27-17** (*E. coli* ST6332) are different in less than 4 alleles to **23-16** (Figure 2). Moreover, results from hybridization and PBRT experiments indicated $bla_{\text{NDM-1}}$ to be associated with a common IncN plasmid. Interestingly, NGS reads and contigs derived from isolates **23-16** and **29-16** and aligned to **pEC6332-T6** without any mismatch (Figures S7 and S8[1]). In contrast, comparable alignments for isolates **23-17**, **25-17**, and **27-17** consistently showed a non-matching region within *dct* (at position 18,143 - 17,643) (data not shown). Based on sequence homologies, we identified an insertion of T*n2*

[1] Supplementary Figures S3 - S23 can be found in Supplementary Data Sheet 2

at this leading to an increased plasmid size of at least 49.8kb (Figure S1[1]). A recovery of the inserted sequence using Sanger was not performed. The corrected plasmid sequence named **pEC6332-T7** was completely covered after aligning NGS reads and assembled contigs of **23-17**, **25-17**, and **27-17**, respectively (Figures S9 and S10[1]).

Results from Southern hybridization and PBRT indicated a *bla*$_{NDM-1}$ associated replicon of the same type and Inc type as **pEC6332-T6** in all *M. morganii* strains **27-16**, **28-16** und **599-16**. Since no validated cgMLST scheme was available, we used a SNP-based analysis (reference: NC_020418.1) revealing only six variations between those isolates. Indeed, aligning NGS reads and assembled contigs of the respective strains lead to full length coverage of the **pEC6332-T6** sequence without any mismatch (Figures S6 and S7[1]).

1.1.3 Plasmids pEC405a-T3 and pEC405b-T3

Using southern hybridization and PBRT *bla*$_{NDM-1}$ was identified on an IncA/C_2 plasmid with a size of approximately 80kb in *E. coli* ST405 strain **128-16** (data not shown). After *de novo* assembly of NGS data derived from this isolate, *bla*$_{NDM-1}$ and IncA/C_2 were located on two separate contigs of 9 and 67kb, respectively. Both and three additional contigs could be perfectly aligned to the IncA/C_2 plasmid **pKP39-T3** (155kb) of *K. pneumoniae* ST39 isolates from this study. The common backbone sequence was flanked by IS*26* IRL and IS*26* IRR elements, respectively. Using PCR a fragment of the size of IS*26* was detected between both ends leading to a circular sequence named **pEC405a-T3** with a final size of 88,530bp. Moreover, aligning initial NGS reads to **pEC405a-T3** revealed a complete coverage (Figures S11 and S12[1]).

A cgMLST considering 2513 core genes revealed a close relationship with only 4 alleles different between the ST405 *E. coli* isolates **128-16** and **130-16** (Figure 2). Additionally, a similar β-lactamase gene pattern was observed in both isolates indicating a common set of accessory genes (Table S1). Based on results from hybridization and PBRT experiments, we suggested the same NDM1-related plasmid shared by both isolates. Indeed, **pEC405a-T3** was completely covered by eight assembled contigs of **130-16**. The alignment showed a region accumulating mismatches in proximity to *kfra* as well as two variations (gaps) within IS*26* elements (data not shown). Sequence homology of the non-matching contig regions revealed an insertion of IS*26* at this site. After considering these variations, read and contig based alignment showed complete reference coverage (Figures S11 and S12[1]). This variation was named **pEC405b-T3.**

1.1.4 Plasmid pPS-T1

Focusing on the *P. stuartii* isolate **21-16**, *bla*$_{NDM-1}$ could be identified on a single contig (42kb, contig A) flanked by one IS*26* IRL and one IS*Kpn18* IRR element, respectively. A corresponding IS*26* IRR element was found on a different contig (contig B) and merged with contig A. The resulting sequence (contig AB) carried IS*Kpn18* IRR at both ends, but lacked any replication sequence. Since results from hybridization experiments and PBRT showed a co-localization of *bla*$_{NDM-1}$ and IncA/C_2, we screened for matching contigs of **21-16**. Indeed, a single contig (111kb; contig C) carried IncA/C_2 but lacked terminal IS*Kpn18* IRL elements. Using an iterative alignment of NGS reads the contig sequence could be reliably extended to IS*Kpn18* IRL elements at both ends. Contigs AB and C were thus merged to a circular sequence. Orientation of Contig B within this construct as well as contig transitions were validated using PCR. Aligning initial NGS reads and assembled contigs to the final sequence named **pPS-T1** revealed full length coverage (Figures S13 and S14[1]). Its final length of 168,682bp is in accordance with results from Southern hybridization experiments and PBRT (data not shown).

1.1.5 Plasmids pKPC-2a, pKPC-2b, and pKPC-2c

In total, 21 KPC-2 positive *K. pneumoniae* strains were identified in this study. After assembling respective NGS data, one *bla*$_{KPC-2}$ carrying contig could be identified for each of those isolates. All contigs shared the same replication sequences (IncFII, IncFIB). By multiple alignments of these contigs a meta-sequence of 165,702bp was generated (data not shown). Notably, all contigs shared the identical sequences in the aligned regions. The meta-sequence exhibited overlapping ends indicating a circular sequence. The site of ring closing has been validated using PCR. The plasmid with a final length of 100,959bp was referred to as **pKPC-2a** thereafter.

For all ST37 isolates (**28-17**, **685-17**, **686-17**, and **1087-16**), the presence of **pKPC-2** was confirmed by aligning reads and contigs (data not shown). No mismatches were detected. An almost complete coverage was achieved when aligning reads and contigs from all ST39 (**210-17**, **692-17**, **696-17**, **1080-16**, **1081-16**, **1083-16**, **1086-16**) and 10 of 11 ST307 isolates (**24-17, 26-17, 211-17, 213-17, 689-17, 690-17-2, 691-17, 693-17, 694-17, 697-17)** to pKPC-2. However, a high number of mismatches within several MGEs and, in case of ST39, *tra* genes could be detected indicating structural variations that were not further analyzed. For the *K. pneumoniae* isolate **22-17** (ST307) we detected a region in pKPC-2 that could not be covered by neither contigs nor reads. This variation has not been further investigated. The plasmid was designated as **pKPC-2a**.

Using PCR, *bla*$_{KPC-2}$ has been also detected in *E. coli* strain **212-17** (ST131). By aligning NGS reads and assembled contigs the pKPC-2 sequence could be largely covered. However, there were several mismatch regions (mostly at alignment ends) and the achieved coverage was quite low indicating structural variations compared to the reference (data not shown). The plasmid was designated as **pKPC-2b**.

1.1.6 Plasmid pEC6332-T3

After assembling NGS-derived reads obtained from **690-17-1** (*E. coli* ST6332), an IncA/C_2 backbone structure including *bla*$_{NDM-1}$ was detected on a single contig (115kb). Interestingly, mapping of this contig to other IncA/C_2 plasmids of this study revealed an almost perfect match to the backbone sequence of **pKP39-T3** (only 2 SNPs within MGEs). The contig was flanked by IS*26* IRL and IS*26* IRR, respectively, which have manually been replaced by one complete IS26 element resulting in a circular sequence. The site of ring closing was validated using PCR. Initial NGS reads and assembled contigs were successfully aligned to the final sequence resulting named **pEC6332-T3** leading to a sufficient coverage (Figures S15 and 16[1]). The final size of 115,265bp was in accordance to results of hybridization and PBRT experiments.

1.2 NGS- and SMRT/ONT-based reconstructions

DNA of selected strains were sequenced using PacBio® Single Molecule Real Time (SMRT) sequencing or Oxford Nanopore Technology (ONT) if

(i.) complete reconstruction of the *bla*$_{NDM-1}$ carrying plasmid solely based on NGS data failed

(ii.) NGS-based reconstruction revealed a new structure of the *bla*$_{NDM-1}$ carrying plasmid.

1.2.1 Plasmid pEC744-T5

Combining NGS and SMRT data, 19 contigs (115 – 2,298,450bp) have been assembled for the *E. coli* strain **24-16** (ST744) one of which encodes both NDM-1 and IncA/C_2 (147 kb). As no complementary sequence regions were found at the end of this contig, iterative alignment of NGS reads has been used for extension (by 244bp in total). The extended contig exhibited complementary sequence ends indicating a ring closing site that has been confirmed using PCR. The resulting circular sequence named **pEC744-T5** comprises 147,541bp which is in accordance with results from Southern hybridization experiments and PBRT. Additionally, NGS reads were aligned to the final sequence resulting in a good coverage (Figure S17[1]).

cgMLST analyses considering 2513 core genes revealed a close genetic relationship between all ST744 *E.coli* isolates of this studies (**24-16**, **25-16**, **26-16**, **129-16**, **131-16**, **133-16**, **459-16**, **687-17**, **688-17**) (maximal difference of 5 alleles; Figure 2). Additionally, a similar β-lactamase gene pattern was detected in all isolates indicating a common set of accessory genes (Supplementary Table 1). Using hybridization experiments and PBRT a 140kb IncA/C_2 plasmid was identified encoding NDM-1 in all isolates. The presence of **pEC744-T5** could be confirmed by aligning respective NGS reads or assembled contigs with only a few mismatches.

1.2.2 iECl-T3 and Plasmid pECl-T3

Since Southern hybridization and PBRT did not detect any plasmid carrying *bla*$_{NDM-1}$ in isolate **460-16** (*E. cloacae*), WGS was performed using NGS and ONT. *De novo* assembly resulted in 3 contigs (1,935 – 5,024,410bp). An IncA/C_2 backbone structure including *bla*$_{NDM-1}$ could be identified on the largest contig representing the bacterial chromosome. MBL gene *bla*$_{NDM-1}$ was located on a 95kb structure flanked by IS*26* on both ends (named **iECl-T3** thereafter).

cgMLST analyses considering 2385 core genes revealed a difference of only four alleles between the *E. cloacae* isolates **460-16** and **629-16**. Additionally, both isolates exhibited a similar β-lactamase gene pattern (Table S1). Interestingly, *bla*$_{NDM-1}$ was detected in bands representing both chromosomal contaminations and an IncA/C_2 plasmid of approx. 90kb using hybridization and PBRT experiments. Thus we suggested that this isolate harbors **iECl-T3** integrated in the bacterial chromosome but additionally as separate circular plasmid with the same sequence but (named **pECl-T3**; 94,919bp). The site of ring closing (resulted from combining both terminal IS*26* elements) has been validated using PCR. NGS reads derived from **629-16** aligned to **pECl-T3** with few mismatches (Figures S18 and S19[1]). Interestingly, was covered by approx. the double number of reads than the adjactant chromosomal regions supporting our suggestion of an additional copy (data not shown).

1.2.3 Plasmid pCF104a-T3

Using NGS and ONT data 5 contigs (3,224 – 5,135,365bp) were assembled for *C. freundii* strain **20-16**. The replication sequences of type IncA/C_2 and IncR as well *bla*$_{NDM-1}$ were identified on the same of 170kb. No complementary sequences were found at the ends of this contig. Thus, we performed iterative alignment of NGS reads to the respective contig sequence resulting in a reliable extension of 301bp in total. The extended contig exhibited complementary terminal sequences which were used to form a circular sequence. The site of circularization has been validated using PCR. Aligning NGS reads and resulting contigs to this sequence revealed full-length coverage with no mismatch (Figures S20 and 21[1]). With a total length of 176.5kb the size of this sequence named **pCF104a-T3** corresponds to results from hybridization experiments and PBRT.

Both *C. freundii* isolates **20-16** and **134-16** showed only a difference of 7 alleles (cgMLST considering 4156 core genes) (Figure 2). Additionally, both isolates exhibited an identical β-lactamase gene pattern indicating a common set of accessory genes. Southern hybridization and PBRT revealed that *bla*$_{NDM-1}$ was associated with an IncN plasmid of 80kb which is significantly shorter than **pCF104a-T3**. Surprisingly, NGS reads and assembled contigs derived from strain **134-16** aligned to the full sequence of **pCF104a-T3**. The only variation detected was an insertion of IS*26* nearby Tn*21*. The inconsistency of these results cannot be explained yet. If the genuine **pCF104a-T3** was divided into two MGEs with IncA/C_2 and IncN replication sequences, respectively, has to be proofed. The variation was referred to as **pCF104b-T3.**

1.2.4 Plasmid pKP15-T2

Isolate 22-16 (*K. pneumoniae* ST15) was isolated from a patient with a travel history. Based on NGS and ONT data, a single contig (126.5kb) encoding IncFIB and NDM-1 was identified. Terminal sequences of this contig were not complementary. Using this contig as a reference for iterative NGS read alignment the contig was reliably extended to 126.7kb. The now existing complementary ends indicated a circular sequence of 126.5kb named **pKP15-T2**. The site of ring closing was confirmed by PCR. Moreover, NGS reads and assembled contigs were aligned to the final sequence to proof full coverage (Figures S22 and 23[1]).
