# Supplementary material for: IS26-Mediated Transfer of blaNDM–1 as the Main Route of Resistance Transmission During a Polyclonal, Multispecies Outbreak in a German Hospital
Source: Front Microbiol. 2019 Dec 17;10:2817. doi: 10.3389/fmicb.2019.02817 (PMC6929489; doi:10.3389/fmicb.2019.02817)
Supplement: Supplementary file 4 [file Data_Sheet_2.pdf]

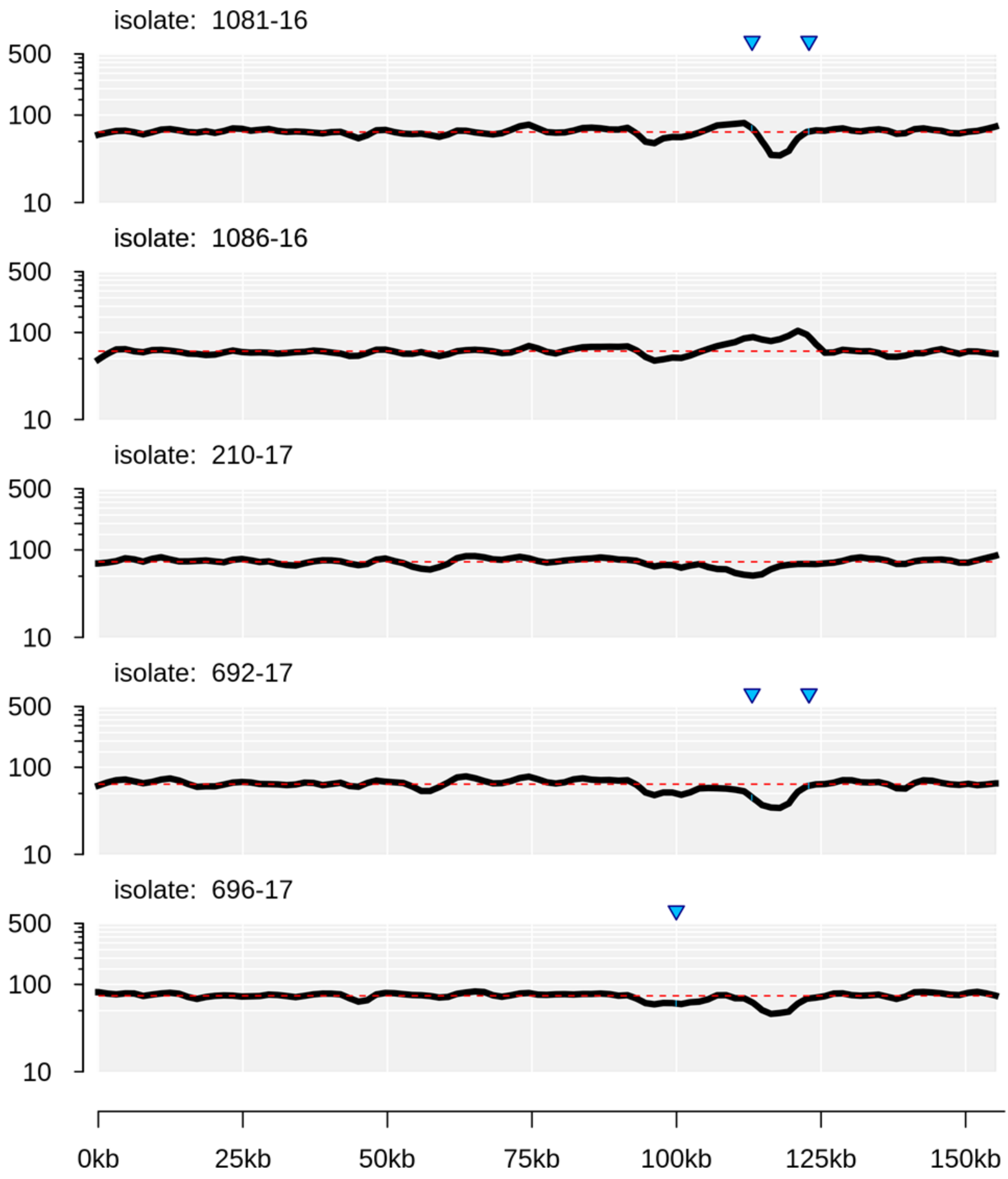

Supplementary Figure S3. Coverage of pKP39-T3 by Illumina reads from different isolates. Trimmed reads were aligned to the final plasmid sequence using Bowtie2 with stringent parameters (see Material and Methods). Shown is the coverage (lowess filter 1/25; log scale; y-axis) across the plasmid sequence (x-axis). Variations are indicated by blue arrows.

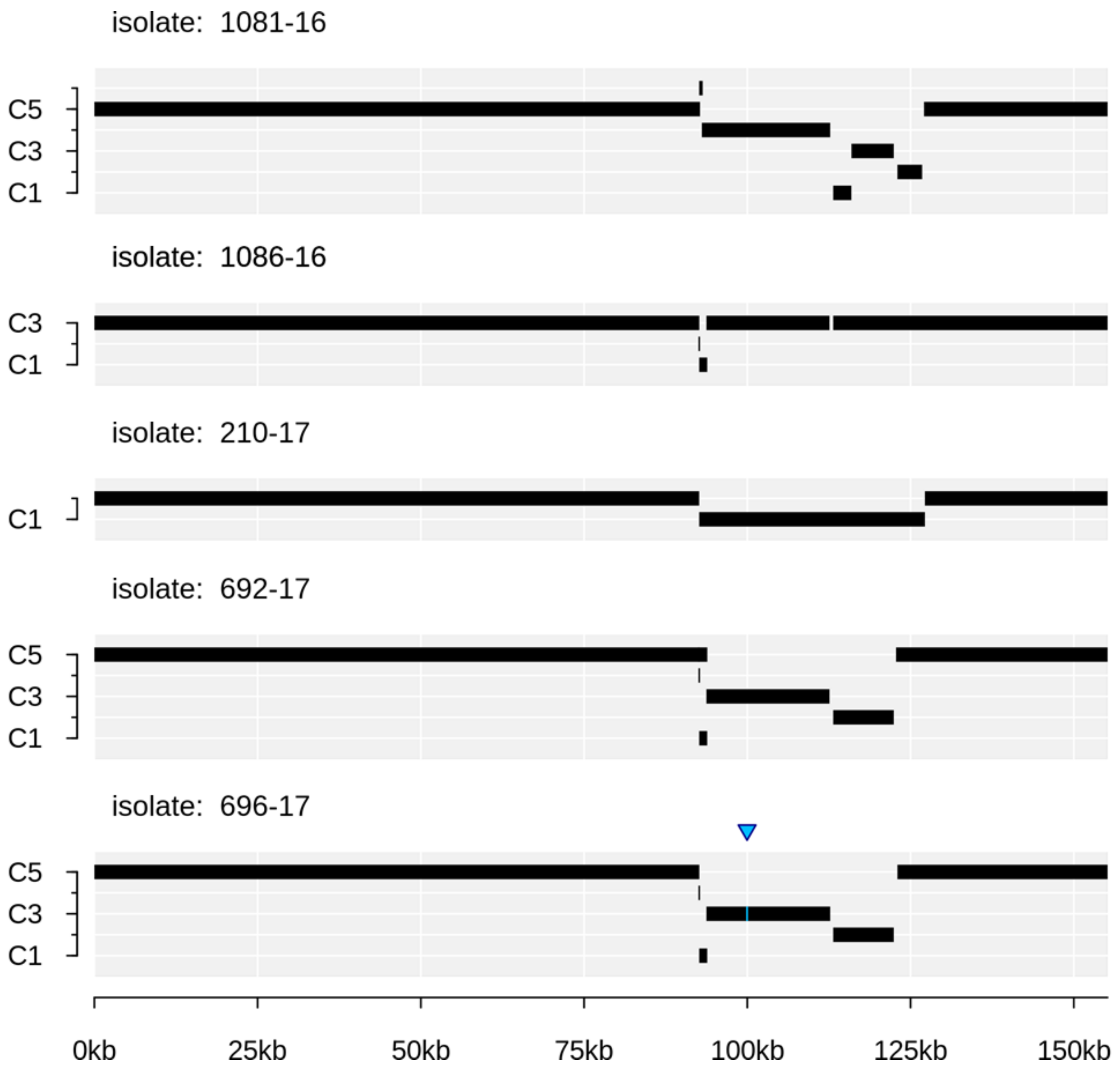

Supplementary Figure S4. Coverage of pKP39-T3 by assembled contigs from different isolates. Illumina reads were assembled using SPAdes. Resulting contigs were aligned to the final plasmid sequence using Mummer3/Nucmer. Shown is the coverage by aligned contigs (y-axis, each line represents one contig) across the sequence (x-axis). Variations are indicated by blue arrows.

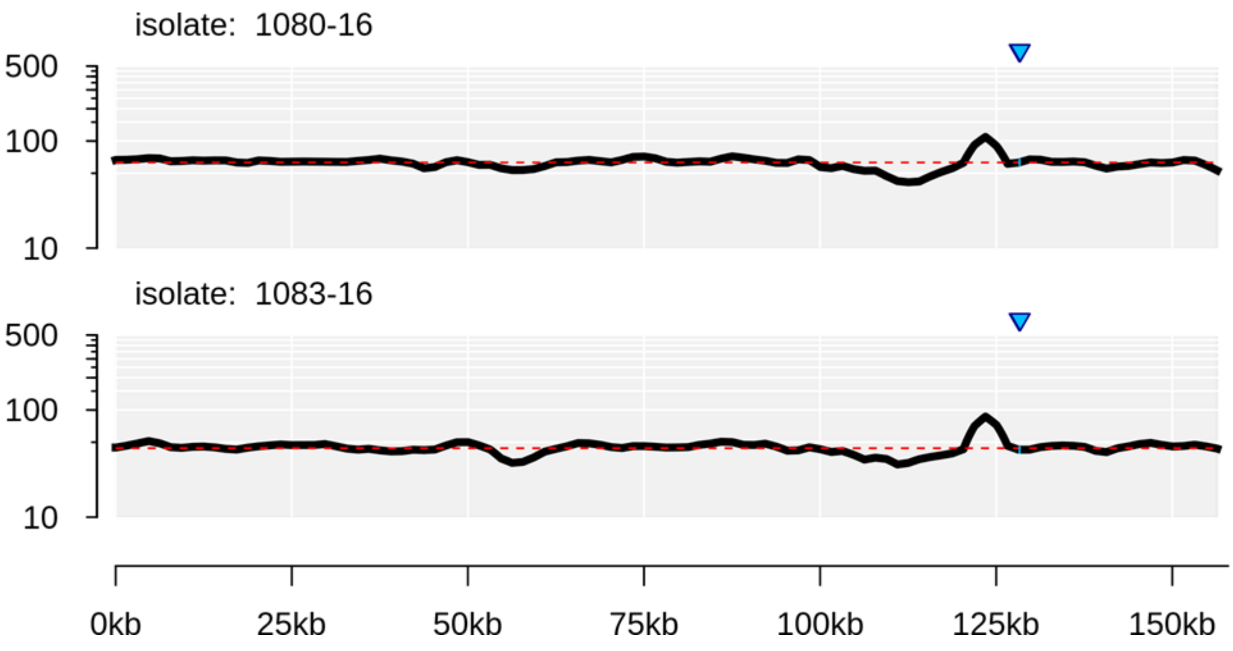

Supplementary Figure S5. Coverage of pKP39-T4 by Illumina reads from different isolates. Trimmed reads were aligned to the final plasmid sequence using Bowtie2 with stringent parameters (see Material and Methods). Shown is the coverage (lowess filter 1/25; log scale; y-axis) across the plasmid sequence (x-axis). Variations are indicated by blue arrows.

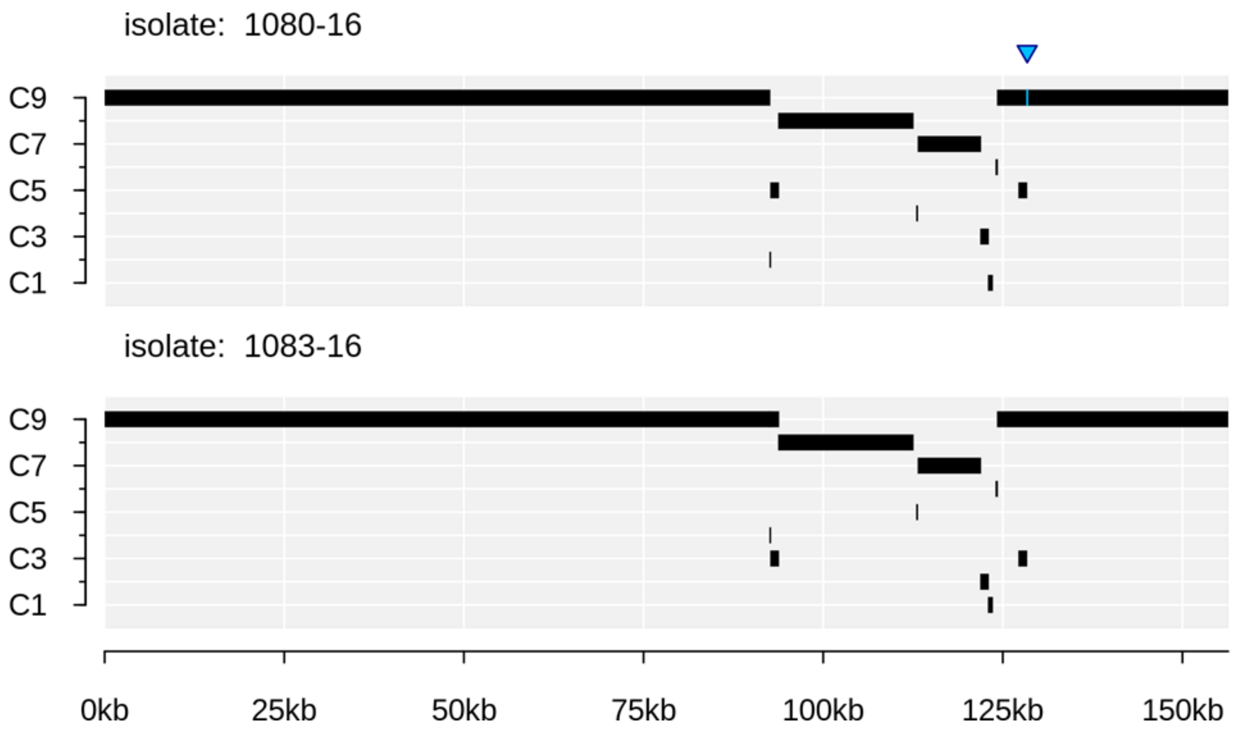

Supplementary Figure S6. Coverage of pKP39-T4 by assembled contigs from different isolates. Illumina reads were assembled using SPAdes. Resulting contigs were aligned to the final plasmid sequence using Mummer3/Nucmer. Shown is the coverage by aligned contigs (y-axis, each line represents one contig) across the sequence (x-axis). Variations are indicated by blue arrows.

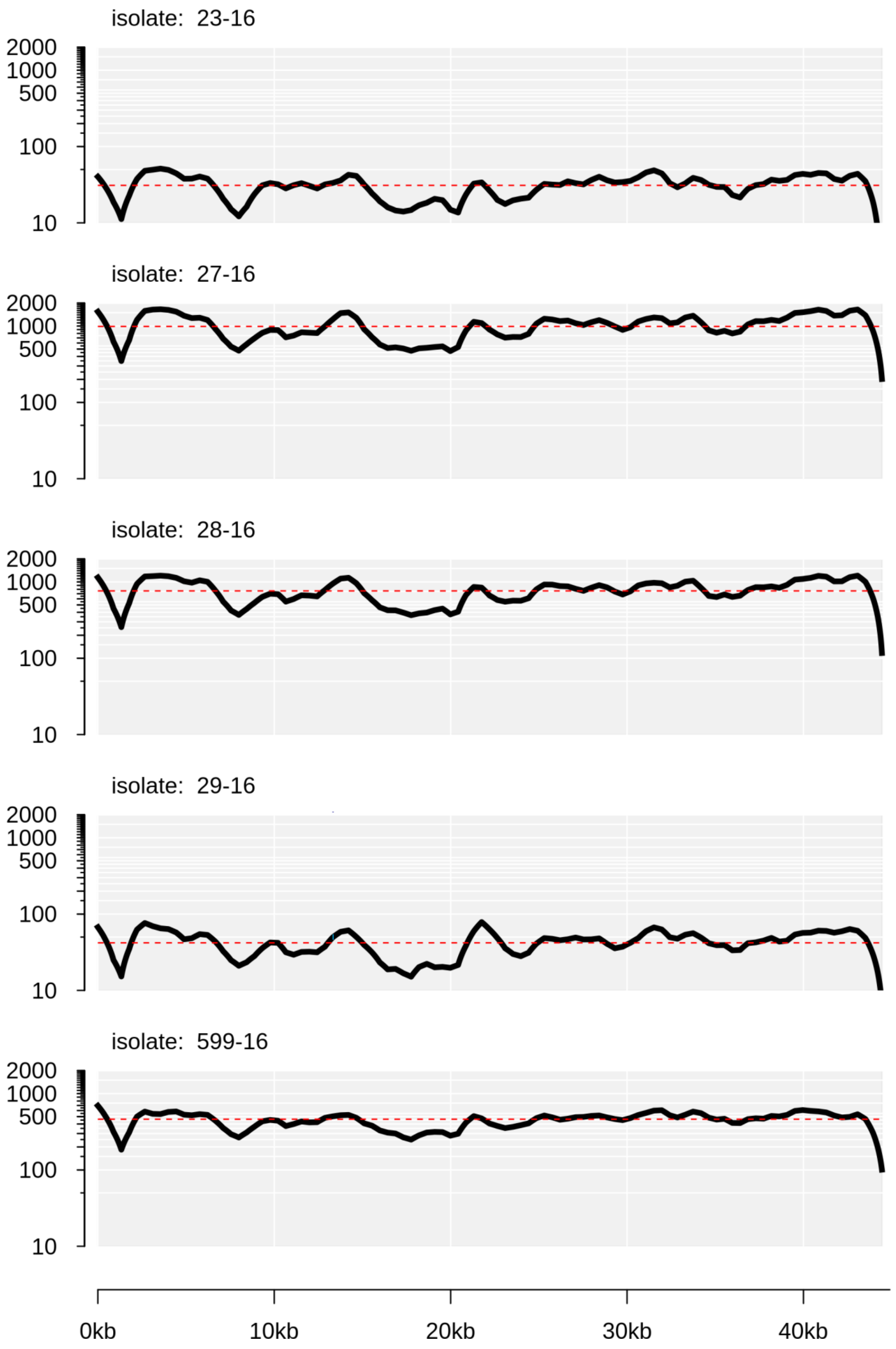

Supplementary Figure S7. Coverage of pEC6332-T6 by Illumina reads from different isolates. Trimmed reads were aligned to the final plasmid sequence using Bowtie2 with stringent parameters (see

Material and Methods). Shown is the coverage (lowess filter 1/25; log scale; y-axis) across the plasmid sequence (x-axis).

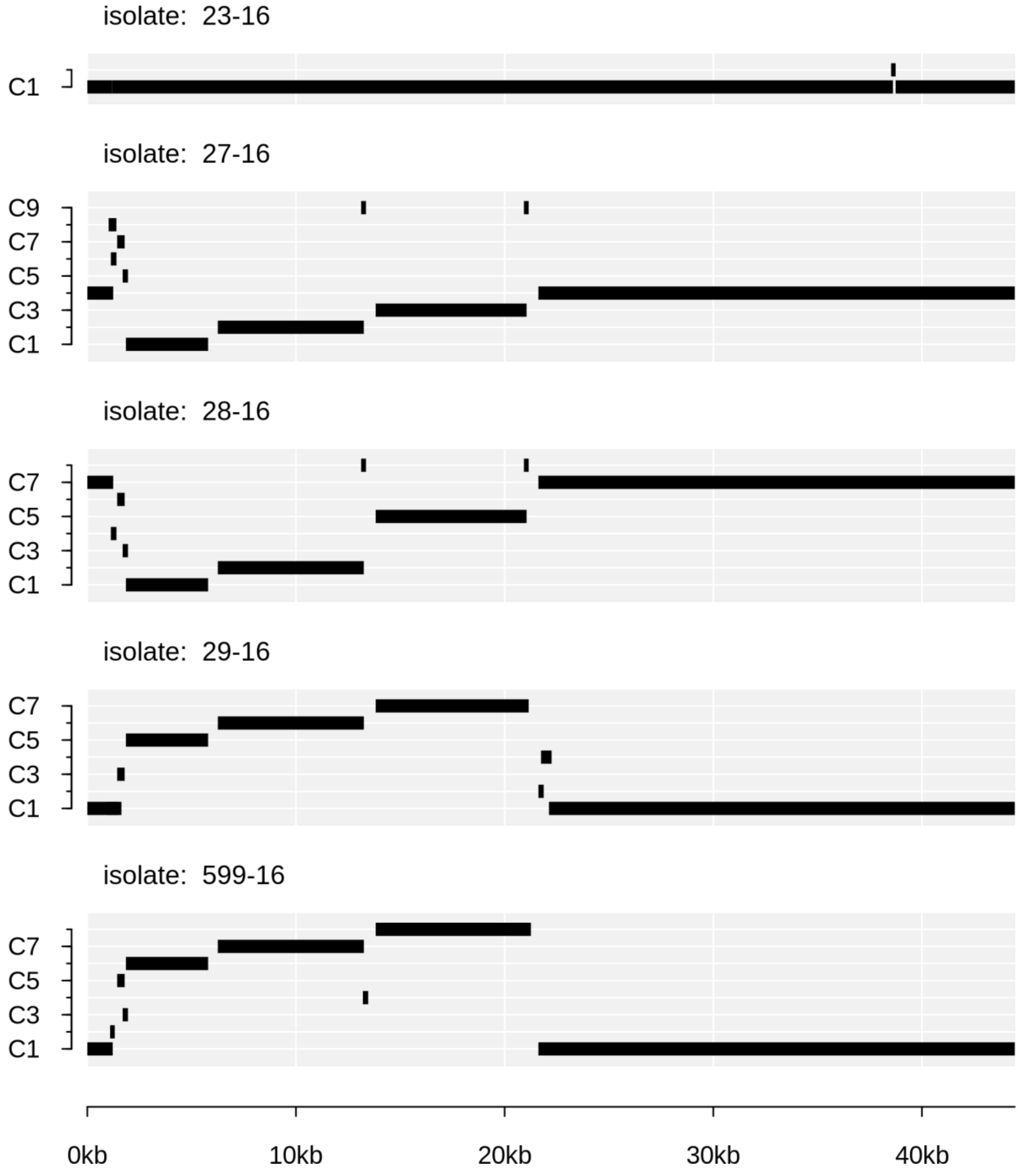

Supplementary Figure S8. Coverage of pEC6332-T6 by assembled contigs from different isolates. Illumina reads were assembled using SPAdes. Resulting contigs were aligned to the final plasmid sequence using Mummer3/Nucmer. Shown is the coverage by aligned contigs (y-axis, each line represents one contig) across the sequence (x-axis).

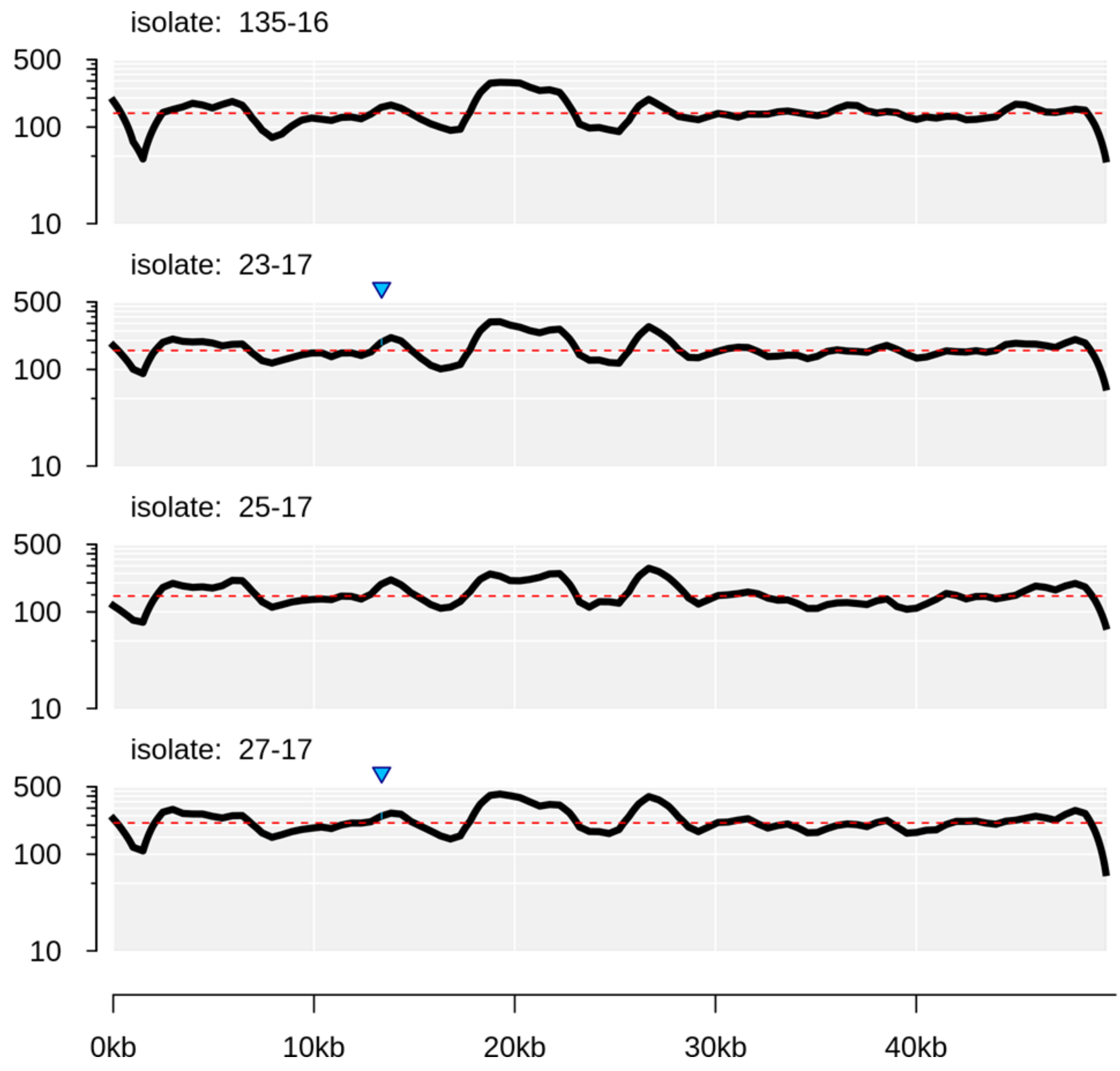

Supplementary Figure S9. Coverage of pEC6332-T7 by Illumina reads from different isolates. Trimmed reads were aligned to the final plasmid sequence using Bowtie2 with stringent parameters (see Material and Methods). Shown is the coverage (lowess filter 1/25; log scale; y-axis) across the plasmid sequence (x-axis). Variations are indicated by blue arrows.

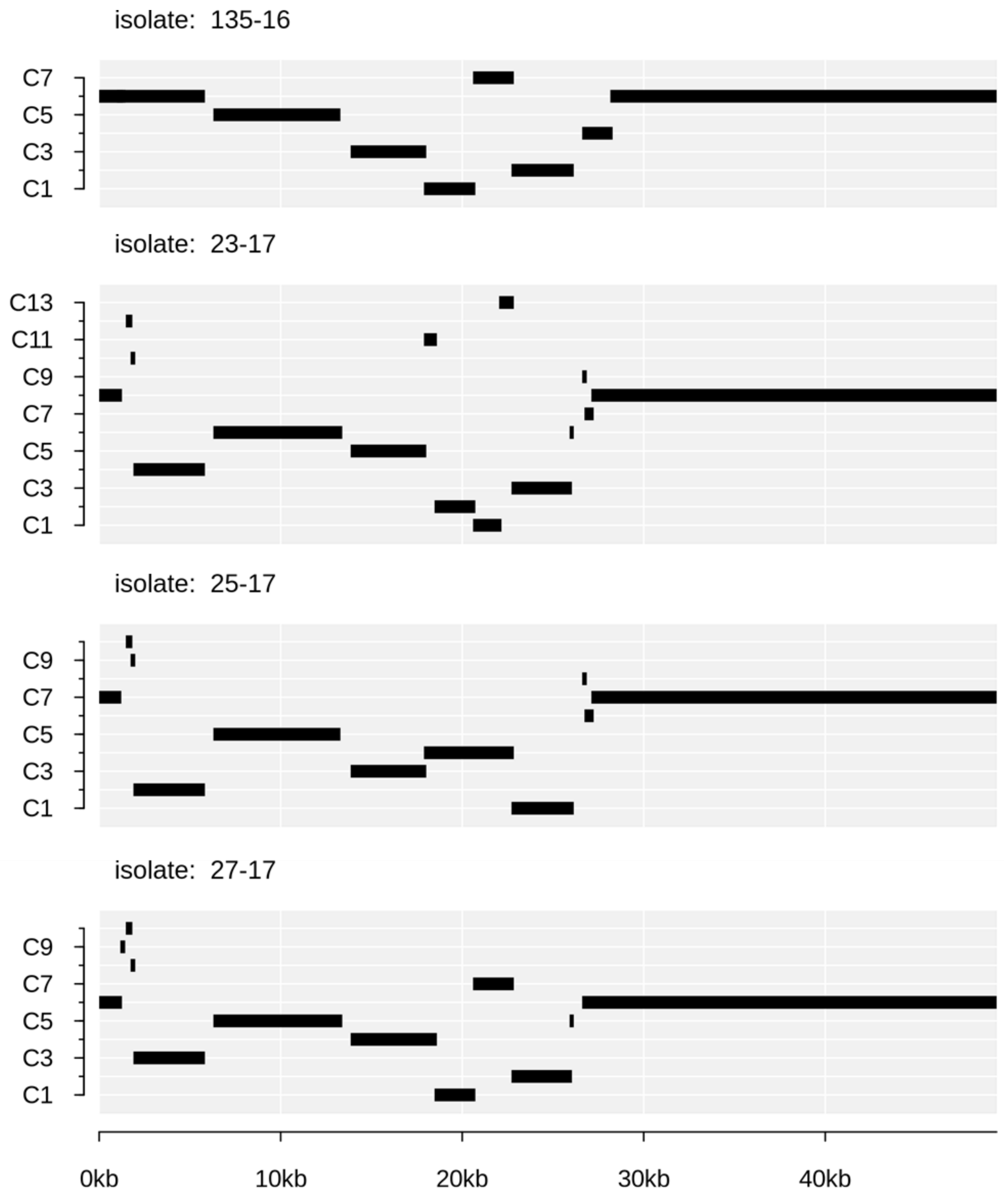

Supplementary Figure S10. Coverage of pEC6332-T7 by assembled contigs from different isolates. Illumina reads were assembled using SPAdes. Resulting contigs were aligned to the final plasmid sequence using Mummer3/Nucmer. Shown is the coverage by aligned contigs (y-axis, each line represents one contig) across the sequence (x-axis).

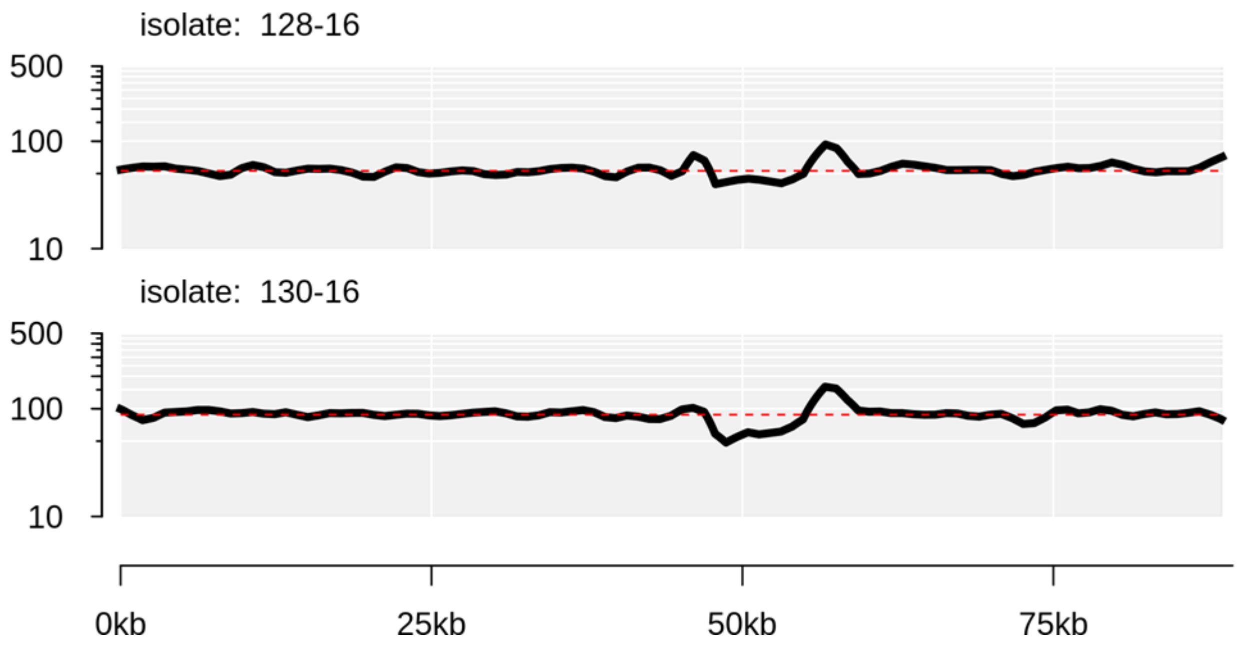

Supplementary Figure S11. Coverage of pEC405a-T3 (top) and pEC405b-T3 (down) by Illumina reads from different isolates. Trimmed reads were aligned to the final plasmid sequence using Bowtie2 with stringent parameters (see Material and Methods). Shown is the coverage (lowess filter 1/25; log scale; y-axis) across the plasmid sequence (x-axis).

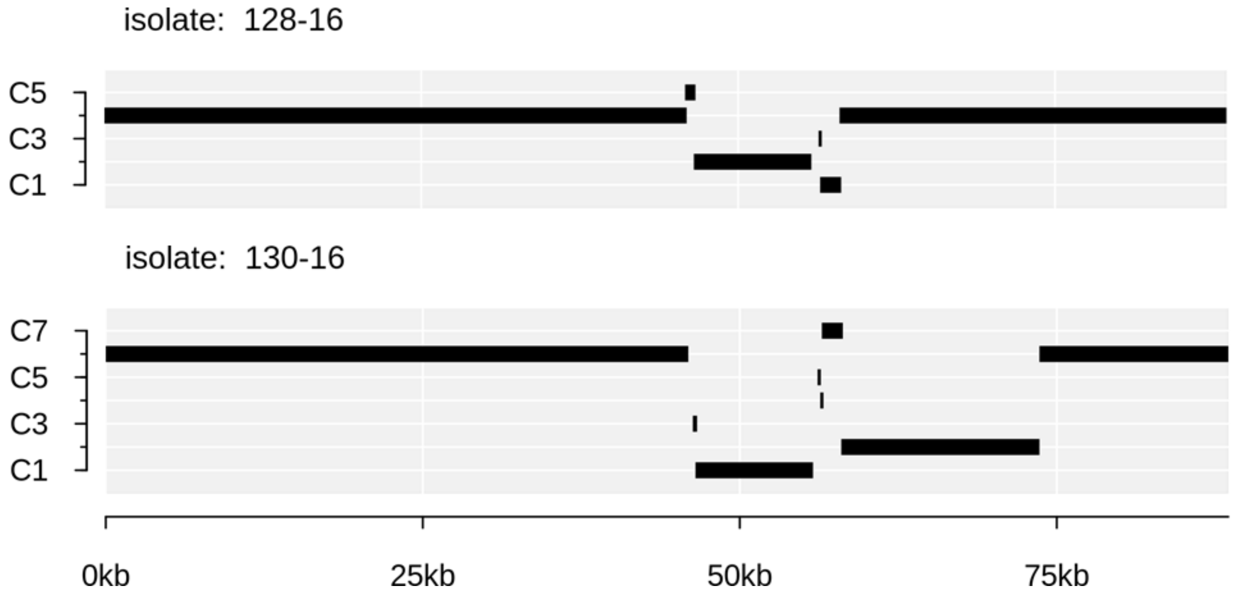

Supplementary Figure S12. Coverage of pEC405a-T3 (top) and pEC405b-T3 (down) by assembled contigs from different isolates. Illumina reads were assembled using SPAdes. Resulting contigs were aligned to the final plasmid sequence using Mummer3/Nucmer. Shown is the coverage by aligned contigs (y-axis, each line represents one contig) across the sequence (x-axis).

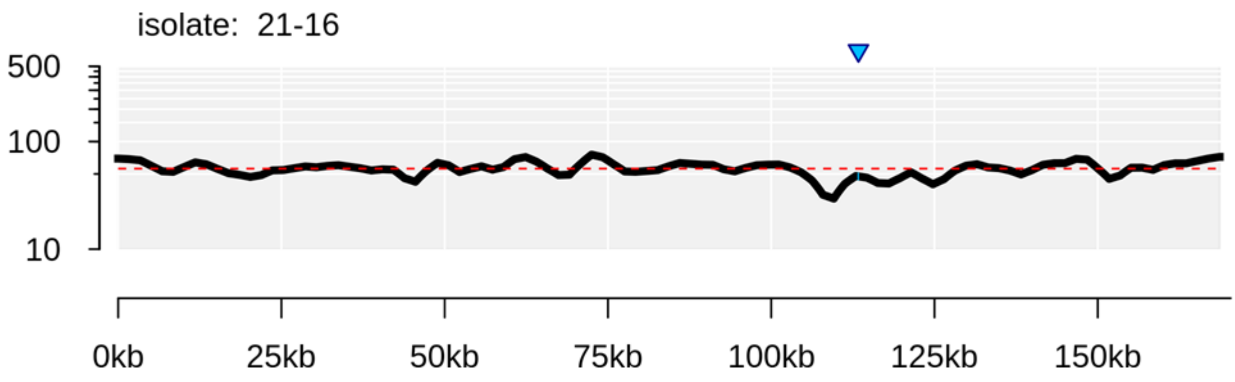

Supplementary Figure S13. Coverage of pPS-T1 by Illumina reads of isolate 21-16. Trimmed reads were aligned to the final plasmid sequence using Bowtie2 with stringent parameters (see Material and Methods). Shown is the coverage (lowess filter 1/25; log scale; y-axis) across the plasmid sequence (x-axis). Variations are indicated by blue arrows.

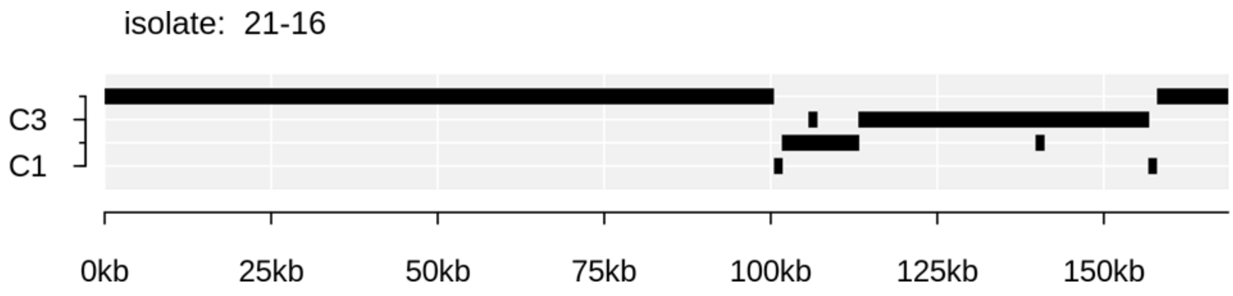

Supplementary Figure S14. Coverage of pPS-T1 by assembled contigs of isolate 21-16. Illumina reads were assembled using SPAdes. Resulting contigs were aligned to the final plasmid sequence using Mummer3/Nucmer. Shown is the coverage by aligned contigs (y-axis, each line represents one contig) across the sequence (x-axis).

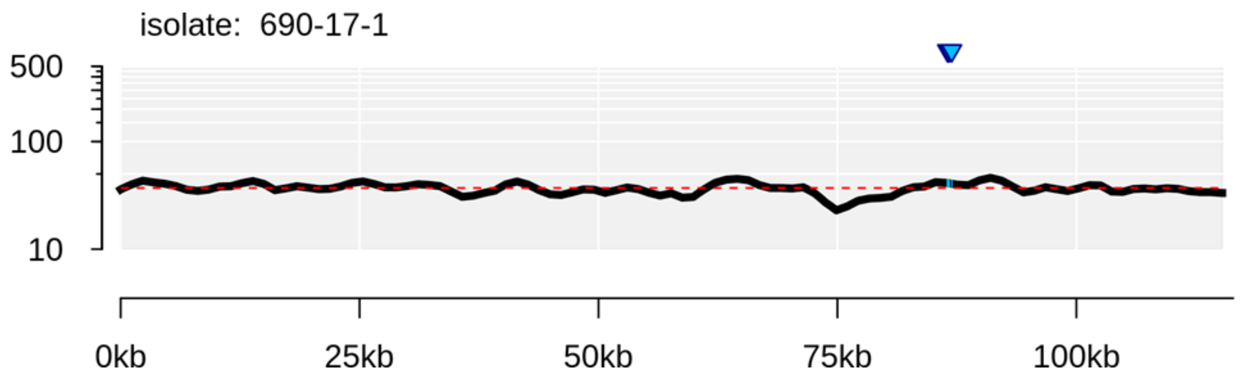

Supplementary Figure S15. Coverage of pEC6332-T3 by Illumina reads of isolate 690-17-1. Trimmed reads were aligned to the final plasmid sequence using Bowtie2 with stringent parameters (see Material and Methods). Shown is the coverage (lowess filter 1/25; log scale; y-axis) across the plasmid sequence (x-axis). Variations are indicated by blue arrows.

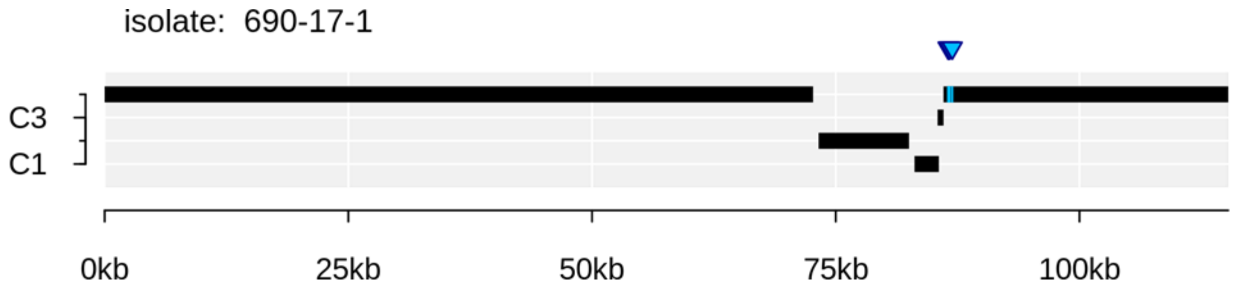

Supplementary Figure S16. Coverage of pEC6332-T3 by assembled contigs of isolate 690-17-1. Illumina reads were assembled using SPAdes. Resulting contigs were aligned to the final plasmid sequence using Mummer3/Nucmer. Shown is the coverage by aligned contigs (y-axis, each line represents one contig) across the sequence (x-axis).

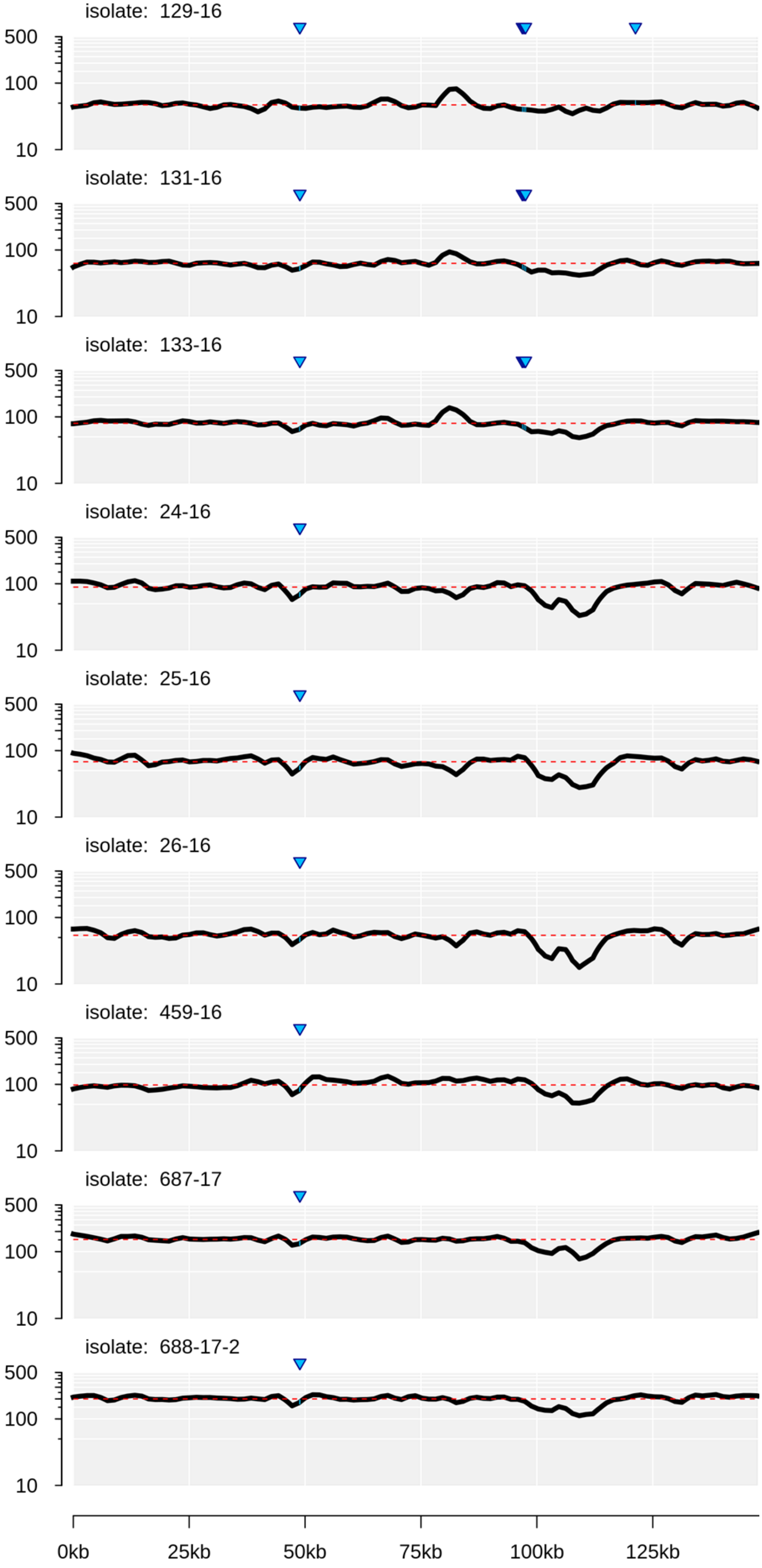

Supplementary Figure S17. Coverage of pEC744-T5 by assembled contigs from different isolates. Trimmed reads were aligned to the final plasmid sequence using Bowtie2 with stringent parameters (see Material and Methods). Shown is the coverage (lowess filter 1/25; log scale; y-axis) across the plasmid sequence (x-axis). Variations are indicated by blue arrows.

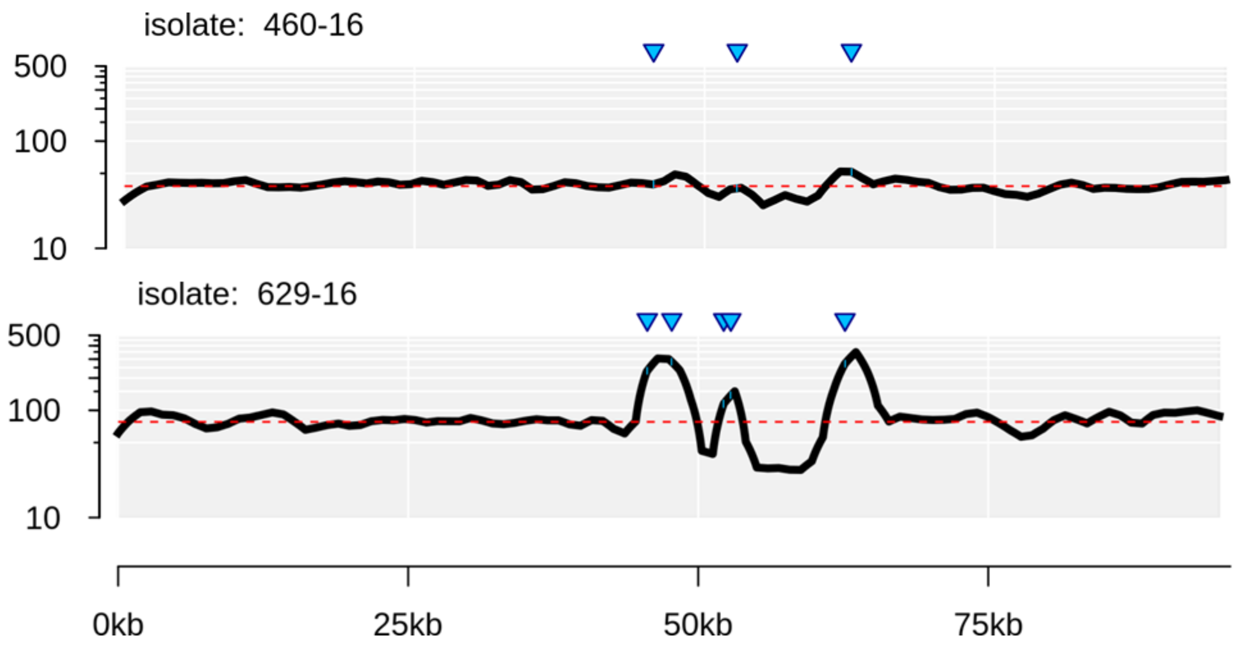

Supplementary Figure S18. Coverage of pECl-T3 by Illumina reads from different isolates. Trimmed reads were aligned to the final plasmid sequence using Bowtie2 with stringent parameters (see Material and Methods). Shown is the coverage (lowess filter 1/25; log scale; y-axis) across the plasmid sequence (x-axis). Variations are indicated by blue arrows.

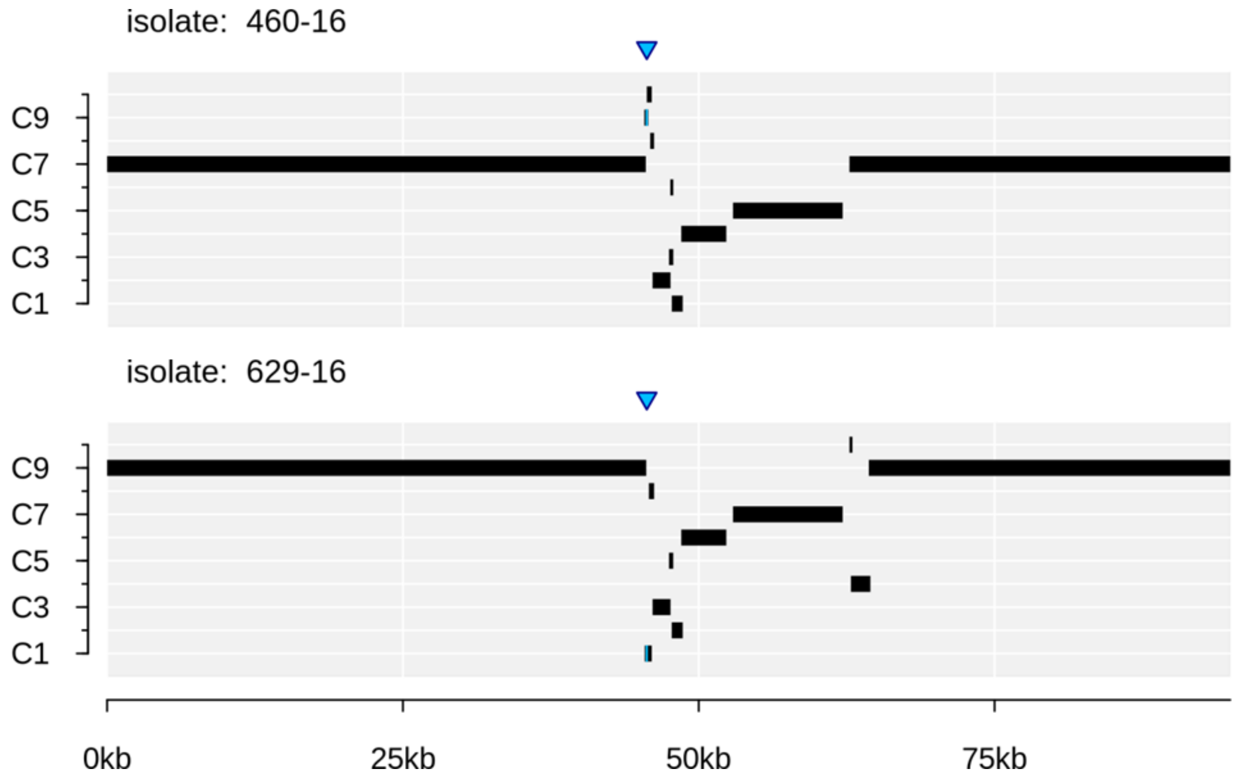

Supplementary Figure S19. Coverage of pECl-T3 by assembled contigs from different isolates. Illumina reads were assembled using SPAdes. Resulting contigs were aligned to the final plasmid sequence using Mummer3/Nucmer. Shown is the coverage by aligned contigs (y-axis, each line represents one contig) across the sequence (x-axis). Variations are indicated by blue arrows.

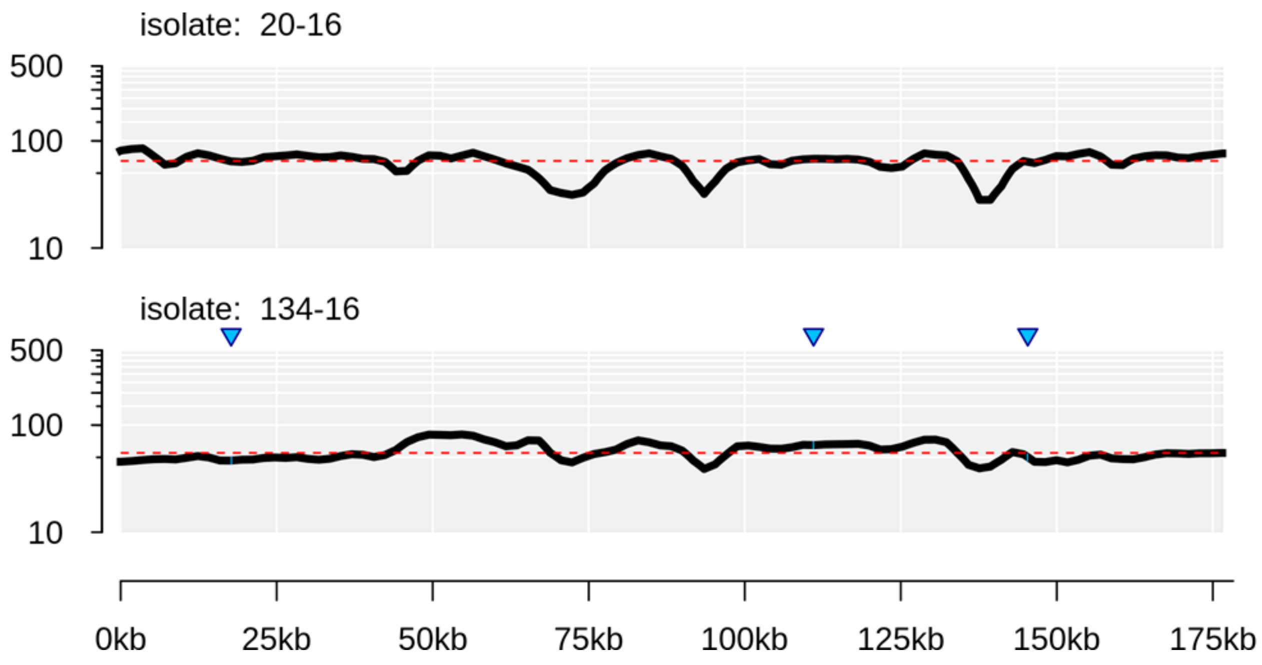

Supplementary Figure S20. Coverage of pCF104a-T3 by Illumina reads from different isolates. Trimmed reads were aligned to the final plasmid sequence using Bowtie2 with stringent parameters (see Material and Methods). Shown is the coverage (lowess filter 1/25; log scale; y-axis) across the plasmid sequence (x-axis). Variations are indicated by blue arrows.

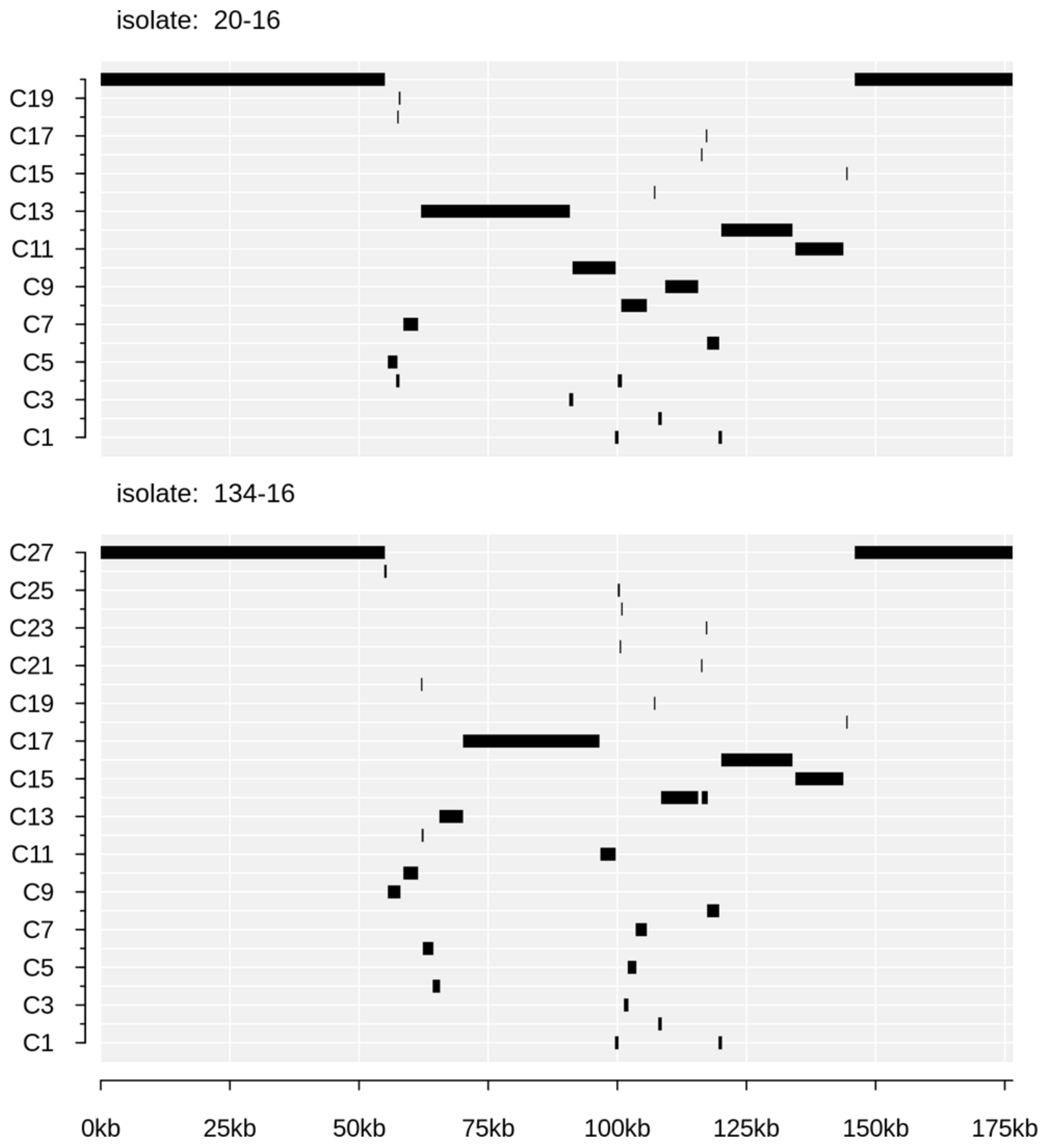

Supplementary Figure S21. Coverage of pCF104a-T3 by assembled contigs from different isolates. Illumina reads were assembled using SPAdes. Resulting contigs were aligned to the final plasmid sequence using Mummer3/Nucmer. Shown is the coverage by aligned contigs (y-axis, each line represents one contig) across the sequence (x-axis).

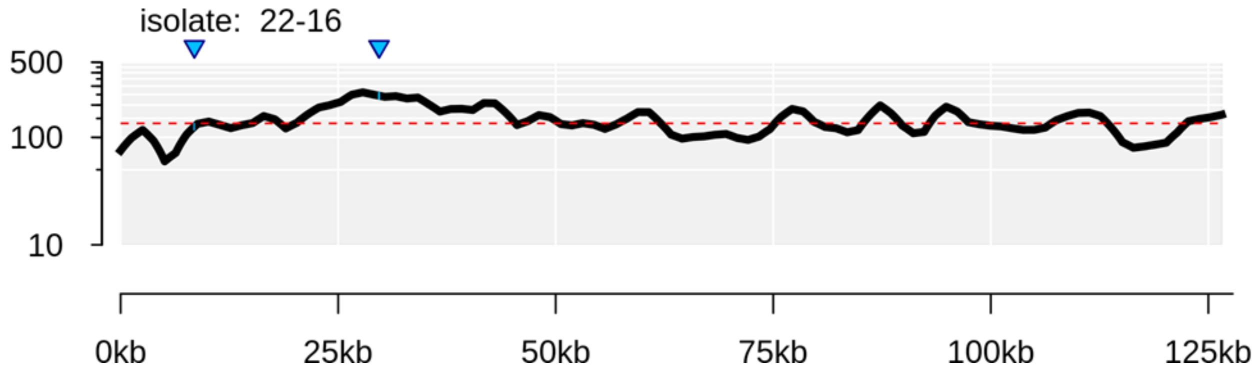

Supplementary Figure S22. Coverage of pKP15-T2 by Illumina reads of isolate 22-16. Trimmed reads were aligned to the final plasmid sequence using Bowtie2 with stringent parameters (see Material and Methods). Shown is the coverage (lowess filter 1/25; log scale; y-axis) across the plasmid sequence (x-axis). Variations are indicated by blue arrows.

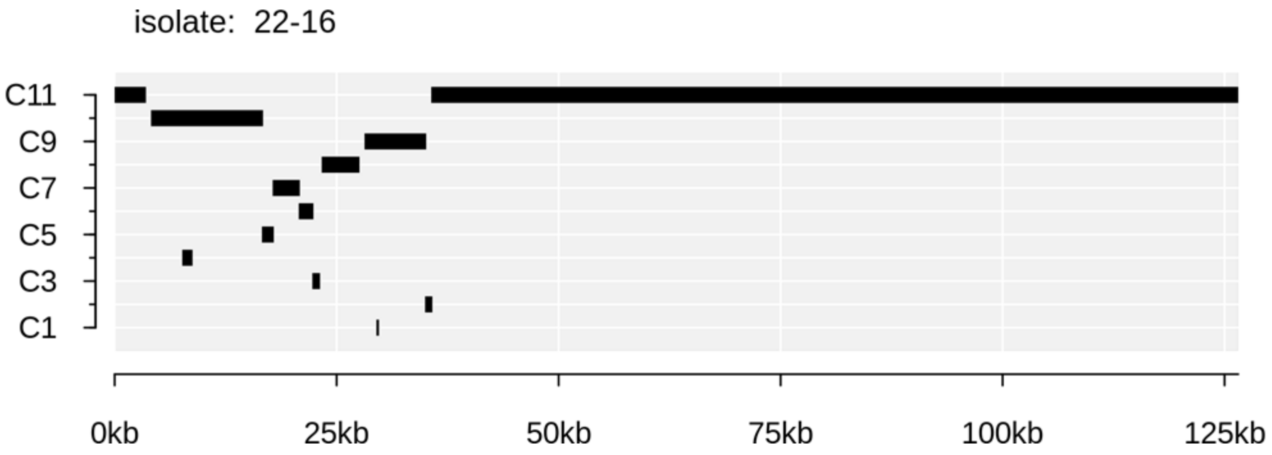

Supplementary Figure S23. Coverage of pKP15-T2 by assembled contigs of isolate 22-16. Illumina reads were assembled using SPAdes. Resulting contigs were aligned to the final plasmid sequence using Mummer3/Nucmer. Shown is the coverage by aligned contigs (y-axis, each line represents one contig) across the sequence (x-axis).
