# Supplementary material for: IS26-Mediated Transfer of blaNDM–1 as the Main Route of Resistance Transmission During a Polyclonal, Multispecies Outbreak in a German Hospital
Source: Front Microbiol. 2019 Dec 17;10:2817. doi: 10.3389/fmicb.2019.02817 (PMC6929489; doi:10.3389/fmicb.2019.02817)
Supplement: Supplementary file 6 [file Table_2.pdf]

Table S2 | Primers used for the validation of plasmid reconstruction (for all PCR reactions performed in this study, an annealing temperature of 52°C was used).

plasmid	Validation of	primer	sequence	product size [bp]
pCF104-T3	pDNA circularization (PCR-based)	pCF104-T3_RS_F	CCTGTCTGGGCACAAGAGTT	686
		pCF104-T3_RS_R	AAATGCTGGCCTCTGATGGT	
pKP15-T2	pDNA circularization (PCR-based)	pKP15-T2_RS_F	TGAGTAATGCGCACAATCCG	391
		pKP15-T2_RS_R	ATCAGCACTGGCCAGATAGC	
pEC744-T5	pDNA circularization (PCR-based	pEC744-T5_RS_F	CAGCCCTGCGTTTTATGAGC	245
		pEC744-T5_RS_R	CGTCTTTCTTGGCCGGAGAT	
pEC405-T3	pDNA circularization (PCR-based)	pEC405-T3_F	TAGGGAGTTTGAGCCGCCTA	2275
		pEC405-T3_R	CCCACTTTAGGTTTGGCAGC	
pECl-T3	pDNA circularization (PCR-based)	pECl-T3_Wdh_F	CCCGTCGCCAAATTCAAGTT	1578
		pECl-T3_Wdh_R	CAGCCACAGCAGAAGGAGAA	
pKP39-T3	pDNA circularization (PCR-based)	pKP39-T3_RS_F	TGTACTGTCGAGGCACTAGC	940
		pKP39-T3_RS_R	GGGTGGGAGCACATCAACC	
pEC6332-T3	pDNA circularization (PCR-based)	RS_pEC6332-T3_F	CCGTACTGCTCCACCATTTCT	1500
		RS_pEC6332-T3_R	ACGCAGTTCTGCAGGTAAAT	
pKPC-2	pDNA circularization (PCR-based)	RS_pKPC-2_F	TTATCCGTGCAGTTCCCTGG	489
		RS_pKPC-2_R	TCTCCGTTCGCCGTTAAAGA	
pEC6332-T6	pDNA circularization (PCR-based)	pEC6332-T6_RS_F	GCCATGCGATTTGTAACCCC	1451
		pEC6332-T6_RS_R	TCCCAGATACCACGAGCTGA	
pPS-T1	Contig orientation 1 (PCR-based)	pPS-T1_F	ACCAGCGTCACGATCAACTT	3220
		pPS-T1_R	CAAAACGTCCCACCAGCTTG	
pPS-T1	Contig orientation 2 (PCR-based)	pPS-T1_2_F	CCAAAGAACGGTCGGAGGAA	3340
		pPS-T1_2_R	GGCTTTTGAAACGCTGGGAC	
pKP39-T3	IS*26* insertion (Sanger Sequencing)	pKP39-T3_IS26_F	CGATACGAGCATTACCAAAGGG	902
		pKP39-T3_IS26_R	CGCAAGCATCTTTTAACGCCT	
pKP39-T4	IS*Kpn26* insertion (Sanger Sequencing)	pKP39-T4_ISK_F1	AGGTGCGACAGTTTCAAAAGC	768
		pKP39-T4_ISK_R1	CCAAAGTGCCACTGATTGCC	
pKP39-T4	IS*Kpn26* insertion (Sanger Sequencing)	pKP39-T4_ISK_F2	CACCATCATTGAGGCACCCA	737
		pKP39-T4_ISK_R2	GGCAAAAAGGCACATGCGAA	
