# Supplementary material for: IS26-Mediated Transfer of blaNDM–1 as the Main Route of Resistance Transmission During a Polyclonal, Multispecies Outbreak in a German Hospital
Source: Front Microbiol. 2019 Dec 17;10:2817. doi: 10.3389/fmicb.2019.02817 (PMC6929489; doi:10.3389/fmicb.2019.02817)
Supplement: Supplementary file 1 [file Image_1.pdf]

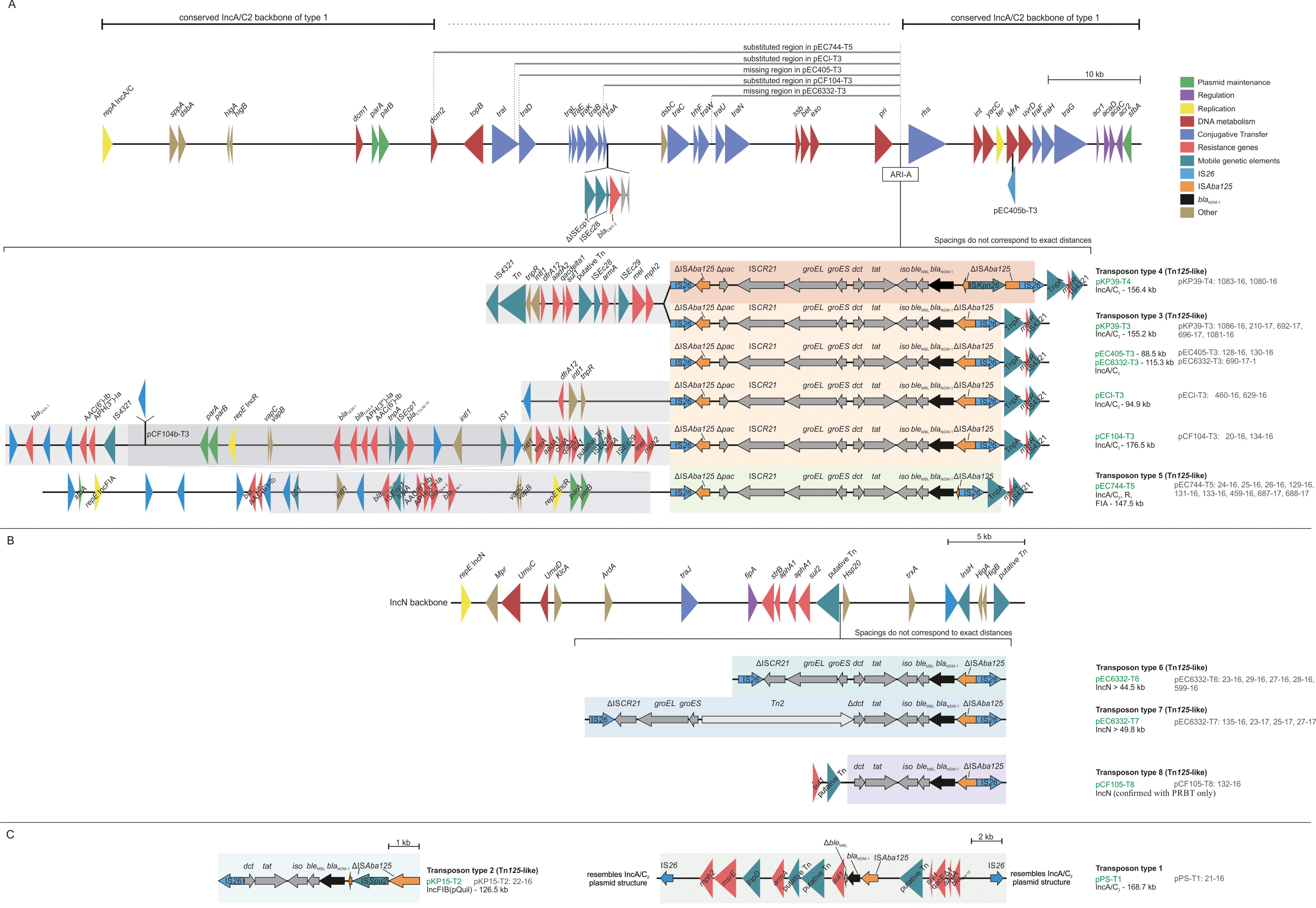


**Supplementary Figure S1 | Genetic organization of $bla_{NDM-1}$ carrying plasmids.** The backbone structures of IncA/$C_2$ and IncN plasmids are shown. Horizontal triangles represent the location, length and orientation of essential ORFs. All IncA/$C_2$ plasmids share a conserved backbone region as described by Harmer and Hall (2015). Missing or variant regions were detected in pEC744-T5, pECl-T3, pEC405-T3, pCF104-T3 and pEC6332-T3. Additionally, the genetic environment of the $bla_{NDM-1}$ associated Tn*125*-like composite transposons is shown in detail. Sequence of pCF105-T8 was not completely recovered. For plasmids belonging to isolates collected from patients with a travel history, only genes in close proximity to $bla_{NDM-1}$ are illustrated.
